# Multifunctional Conductive Polymer Nanocomposite for Intelligent Pesticide Sensing: Trends, Challenges, and Future Prospects

**DOI:** 10.3390/molecules31173133

**Published:** 2026-09-07

**Authors:** Ulfiatun Nisa, Budi Riza Putra, Rayyan Azzahra Hidayat, Dyah Iswantini, Wulan Tri Wahyuni

**Affiliations:** 1 Department of Chemistry, Faculty of Mathematics and Natural Sciences, IPB University, Bogor 16680, Indonesia; ulfiatunnisa1@gmail.com (U.N.); rayyanzhra@gmail.com (R.A.H.); 2 Research Center for Nanotechnology System, National Research and Innovation Agency (BRIN), South Tangerang 15315, Indonesia; budi.riza.putra@brin.go.id; 3 Tropical Biopharmaca Research Center, IPB University, Bogor 16680, Indonesia

**Keywords:** conductive polymer composites, organophosphate pesticides, electrochemical sensors, molecularly imprinted polymers, nanocomposites

## Abstract

Widespread use of organophosphate pesticides (OPPs) raises environmental, food safety, and public health concerns, and drives demand for rapid, sensitive, portable analytical technologies. We reviewed conductive polymer composite (CPC)-based electrochemical OPP sensors, focusing on the structure–performance relationships of representative polymers, polyaniline (PANI), polypyrrole (PPy), polythiophene (PTh), and poly(3,4-ethylenedioxythiophene) (PEDOT), and their hybrids with carbon nanomaterials, metal nanoparticles, metal oxides, MXenes, and metal–organic frameworks. We provided a critical comparison of conductive polymer systems, composite architectures, and enzymatic, molecularly imprinted polymer-based, and direct electrocatalytic sensing strategies, highlighting how material selection influences performance and applicability. Reported CPC-based sensors achieved limits of detection from micro- to femtomolar and successful application in real environmental and food samples; however, these results illustrate the capabilities achieved by selected high-performing reported platforms rather than the overall performance of CPC-based sensors. Despite these advances, reproducibility, long-term operational stability, matrix effects, sensor variability, and field implementation still limit deployment. We discussed future directions to address these challenges, including AI-assisted data analysis, standardised validation, portable field platforms, and sustainable fabrication. Rather than identifying a universally superior platform, this review emphasises rational material selection and composite design to guide CPC sensor development and translation for environmental monitoring and food safety applications.

## 1. Introduction

The expanding global population has increased the demand for food and agricultural products, prompting efforts to increase agricultural yields through the application of fertilisers and pesticides in farming [1,2]. Pesticides are crucial in modern agriculture because they improve crop yields and quality while reducing the effort required for pest control. Studies indicate that their absence could lead to significant reductions in production: 78% in fruits, 58% in vegetables, and 32% in cereals [3]. Organophosphate compounds (OPs) are especially effective insecticides, herbicides, and fungicides. These compounds, derived from phosphoric or phosphorothioic acid, account for approximately 2 million tonnes of global pesticide use annually, with Europe, the United States, and India being the main consumers [4,5]. Due to their widespread application in agriculture and broad-spectrum pesticidal activity, OPs are among the most commercially important pesticides worldwide. Agricultural pesticide use reached 3.70 million tonnes of active ingredients in 2022, representing a 4% increase compared with 2021, a 13% increase over the previous decade, and approximately double the level reported in 1990 [6]. The increasing global dependence on pesticides reinforces growing public concern regarding environmental contamination, food safety, and human exposure. Pesticide poisoning is a major public health issue worldwide, with an estimated 150,000 deaths annually. Organophosphorus and carbamate insecticides account for a large fraction of severe poisoning cases, particularly in low- and middle-income countries [7]. These findings emphasise the urgent need for rapid, sensitive, reliable analytical technologies for monitoring pesticide residues in environmental and food matrices.

Organophosphates act by inhibiting acetylcholinesterase (AChE), an enzyme essential for breaking down acetylcholine (ACh) in the nervous system. This inhibition causes ACh to accumulate at nerve junctions, leading to nervous system overstimulation, paralysis, seizures, and death [8]. Kinetic studies on organophosphate pesticides (OPPs) have shown varying inhibitory effects on AChE across species, indicating varying toxicities among these compounds [9]. The OPPs methamidophos and fenamiphos are especially effective AChE inhibitors, which raise significant public health concerns owing to their widespread agricultural use [10,11]. AChE inhibition can trigger a peripheral cholinergic crisis and status epilepticus, which, if not controlled, can lead to respiratory failure and death [12]. Furthermore, increasing evidence suggests that OPP exposure induces oxidative stress, endocrine disruption, and multiorgan pathological effects that contribute to long-term health risks independent of acute cholinergic toxicity [13]. Therefore, extensive use of OPPs also raises broader health concerns within agricultural ecosystems.

Beyond their direct effects on human and animal health, OPPs disturb environmental stability by disrupting microbial communities vital for nutrient cycling and plant health [14]. Persistent residues in agricultural soils can reduce microbial diversity and soil fertility over time, thereby risking long-term crop productivity [15]. Moreover, OPPs often contaminate aquatic ecosystems through runoff, leaching, or improper disposal [16]. These pollutants significantly threaten aquatic life, including fish and invertebrates, by impairing their physiological and reproductive functions [17]. Contaminated irrigation water spreads pesticide residues further across farmland, increasing their environmental impact [16]. The widespread presence of OPPs highlights the urgent need for effective management strategies to mitigate their negative impacts on agricultural sustainability [18]. In addition, OPP residues accumulate in crops such as fruits, vegetables, and grains, which is a serious concern [19]. Without proper monitoring, these residues can cause harmful dietary exposure. Consequently, developing rapid, reliable, and sensitive methods for detecting OPP residues is increasingly necessary for environmental monitoring and food safety assessment [20].

Various analytical methods have been used to detect OPPs in different sample matrices, including gas chromatography [21,22,23], liquid chromatography [24,25,26], and optical-based sensing [27,28,29]. These methods offer high sensitivity and specificity, but they often carry high equipment costs, the need for large volumes of reagents and solvents, labour-intensive sample preparation, and the requirement for skilled operators. Furthermore, these techniques are complex and time-consuming, restricting their practicality for routine or on-site applications [19]. These limitations have generated interest in developing alternative sensing platforms that can provide rapid, portable, and affordable OPP residue detection. Electrochemical sensing has emerged as a potential alternative. Several recent review articles have summarised electrochemical pesticide sensors, conductive polymers, nanomaterial-based sensing platforms, or individual recognition strategies, but comprehensive, critical evaluation specifically focused on conductive polymer composite (CPC)-based electrochemical sensors has been limited. Previous reviews have emphasised material development or analytical performance, with less attention given to systematically comparing different conductive polymer systems, composite architectures, and recognition strategies and their influences on electrochemical sensing performance and practical applicability. To address this gap, this review critically compares representative conductive polymer systems and CPC architectures, evaluates enzymatic, molecularly imprinted polymer (MIP)-based, and direct electrocatalytic sensing strategies, and discusses the remaining challenges and future opportunities for the practical translation of CPC-based electrochemical sensors for OPP pesticide monitoring.

Electrochemical methods are attractive alternatives owing to their inherent benefits, which include cost-effectiveness, simplicity, portability, rapid response, and the ability to perform real-time, in situ measurements [30]. In contrast, chromatographic techniques, such as GC, GC-MS, and HPLC, although more established, require expensive instrumentation, complex sample preparation, and a separation step that imposes analysis time, which precludes real-time monitoring [31]. Some non-enzymatic electrochemical platforms have achieved response times as short as 10 min while retaining high selectivity [32]. These methods also reduce chemical use and enable miniaturisation and integration into portable devices, making them suitable for field-based applications [33,34]. Recent progress in electrode materials and sensor fabrication has lowered OPP detection limits in some non-enzymatic designs down to the femtomolar (10^−15^ M) range [31,35,36]. Sensor technology developments, such as nanomaterial-based electrodes, have further enhanced these techniques, increasing their feasibility for on-site applications in both agricultural and environmental monitoring [37,38].

CPCs have become promising materials for electrode modification. Conductive polymers (CPs) offer excellent electrical conductivity, reversible redox activity, and adjustable physicochemical properties that benefit electrochemical sensing applications [39]. CPs such as polyaniline (PANI), polypyrrole (PPy), and poly(3,4-ethylenedioxythiophene) (PEDOT) possess conjugated π-electron backbones that enable efficient electron transport and fast charge-transfer processes [40]. This unique structural backbone allows these polymers to function as both efficient electrochemical transducers and versatile platforms for the stable immobilisation of biomolecules. Moreover, their high surface functionality and biocompatibility make them ideal platforms for biomolecule immobilisation and electrochemical transduction. Unlike traditional electrode materials, CPs can be synthesised directly on electrode surfaces using electrochemical or chemical polymerisation, enabling precise control over film morphology, porosity, thickness, and surface chemistry [41]. Additionally, CP systems such as PEDOT:polystyrene sulfonate (PEDOT:PSS) exhibit excellent solution processability and mechanical flexibility, facilitating the development of portable and wearable sensing devices for real-time pesticide monitoring [42]. Owing to these benefits, CP-based electrodes have become attractive platforms for the sensitive and selective detection of OPP residues in environmental and food samples.

Current research emphasises the development of multi-component composite films that integrate CPs such as PEDOT:PSS with nanomaterials, such as graphene or carbon nanotubes, to improve the electrical conductivity and mechanical robustness. These composite architectures have been widely adopted for flexible, portable electrochemical sensing platforms and wearable bioelectronic devices, demonstrating the versatility of PEDOT:PSS for real-time monitoring applications [42,43]. Furthermore, the move toward printable and recyclable architectures ensures that these sensing platforms remain cost-effective and environmentally sustainable for large-scale environmental monitoring [44]. Advances in post-treatment techniques, such as secondary doping with polar solvents or mild acids, continue to optimise these materials by improving PEDOT crystallinity and removing the insulating PSS to promote long-term reliability under humid or harsh environmental conditions [45,46].

Despite these advantages, CPC-based electrochemical sensors continue to face important limitations. CPCs have substantially enhanced electron transfer and sensing performance; however, many reported platforms remain constrained by limited long-term operational stability, sensor-to-sensor reproducibility, matrix interference in complex environmental and food samples, and inconsistencies arising from material synthesis and electrode fabrication. These challenges often complicate direct comparisons among reported sensors and hinder the translation of promising laboratory-scale performance into reliable field applications. These issues are frequently acknowledged in individual studies; however, they are often discussed separately by material type or sensing strategy rather than critically evaluated within a unified framework. Therefore, a comprehensive assessment that systematically compares CP systems, composite architectures, and sensing mechanisms is needed to identify the factors governing analytical performance and practical applicability. This review provides a comprehensive overview of recent advances in CPC-based electrochemical sensors for OPP detection. The discussion critically examines representative CPs, including PANI, PPy, PEDOT, PEDOT:PSS, and PTh, together with their integration with carbon nanomaterials, metal nanoparticles, metal oxides, MXenes, molecularly imprinted polymers, and other emerging functional materials. Both enzymatic and non-enzymatic sensing strategies are evaluated with emphasis on the relationships among CP composition, composite architecture, sensing mechanisms, and analytical performance. In addition, recent developments in portable, paper-based, wearable, and flexible sensing platforms are discussed alongside the remaining challenges related to reproducibility, operational stability, matrix effects, fabrication consistency, and practical field implementation. Finally, future prospects are presented by highlighting emerging opportunities in artificial intelligence-assisted electrochemical analysis and sustainable sensor fabrication for next-generation OPP pesticide monitoring.

## 2. OPPs: Properties and Toxicity

**Figure 1:**
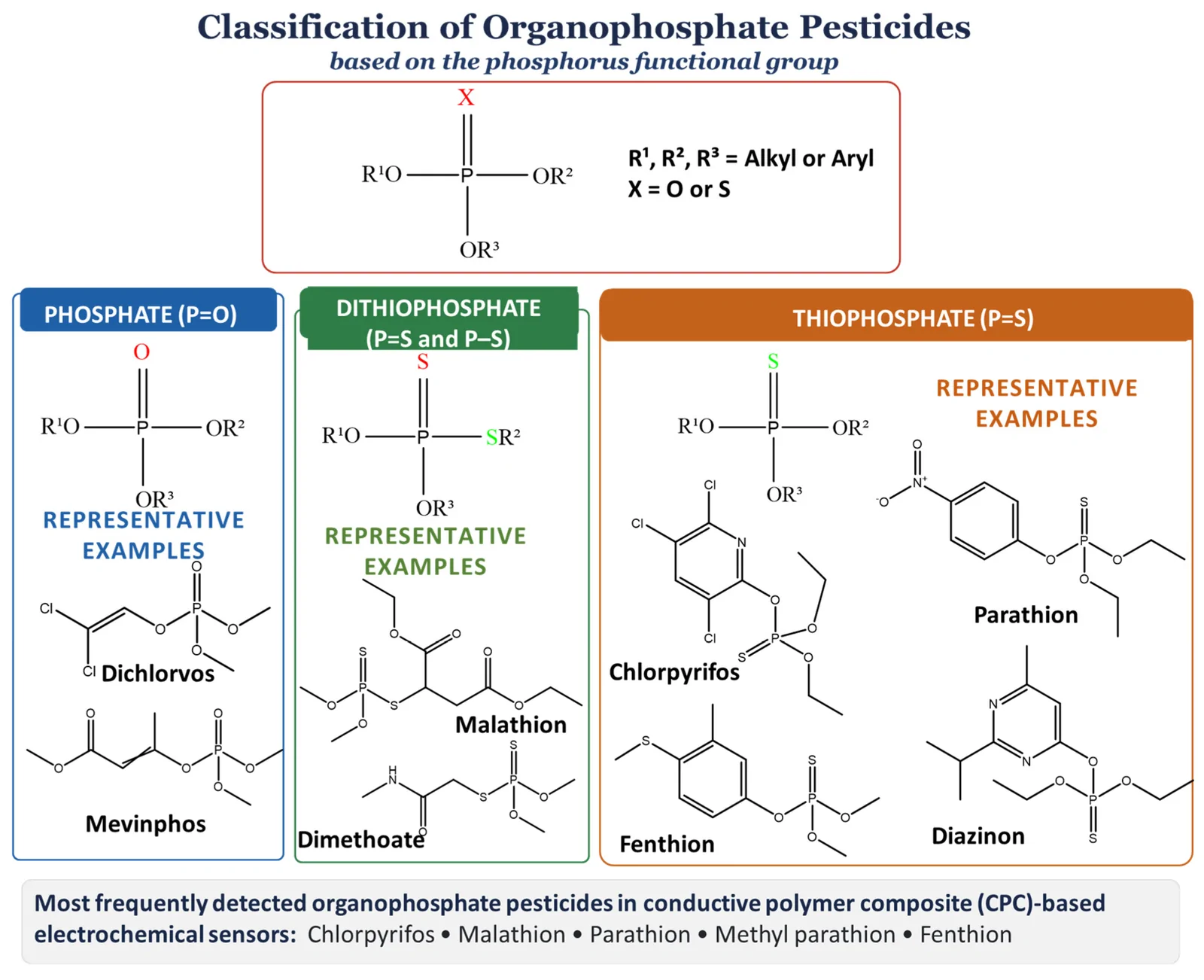
Structural classification of representative organophosphate pesticides (OPPs) by phosphorus functional group. Classification scheme adapted from Saad et al. [16]; chemical structures prepared by the authors using ChemDraw (Free Pro 8.0).

OPPs are among the most widely used insecticide classes in modern agriculture due to their broad-spectrum activity, high effectiveness, and, compared to many organochlorine pesticides, their more rapid degradation. Their widespread use has played a significant role in crop protection and boosting agricultural productivity. However, although most OPPs are less environmentally persistent than organochlorine pesticides, their faster degradation does not eliminate the risk. Continuous intensive application, repeated environmental inputs, and toxic transformation products can still result in accumulated pesticide residues in food products, soil, and water, posing significant risks to human health and ecosystems [47,48,49]. Chemically, OPPs are derived from phosphoric, phosphonic, phosphorothioic, and phosphonothioic acids, usually in the form of esters, amides, or thiol derivatives [50]. They can be broadly classified into phosphate, thiophosphate, and dithiophosphate compounds, as shown in Figure 1. Their properties include moderate water solubility, high lipid affinity, and, in most cases low volatility, which influence their environmental behaviour, bioaccumulation potential, and persistence in agricultural environments [51]. While OPPs lack the extreme longevity of legacy chemicals, uninterrupted application ensures their toxic byproducts accumulate, threatening water safety and food security [47,52].

### 2.1. Chemical Classifications

The versatility of OPPs stems from the structural diversity of their phosphorus-containing functional groups. Their primary classification is based on whether oxygen or sulfur is substituted at the phosphorus centre [16]. Understanding these variations is essential, as sulfur, in the form of thiono (P=S) or thiolo (P-S) groups, often requires metabolic activation (desulfuration) in the target organism [16,53,54], whereas oxygen usually does not.

Three primary OPP categories are defined by their structures [55]:

1. Phosphates: These compounds feature a P=O (phosphoryl) bond and are characterised by higher direct reactivity. Because they possess the active phosphoryl group, they are typically potent AChE inhibitors with no need for prior metabolic conversion in the target organism.
2. Thiophosphate: These compounds contain a P=S (thiono) bond. They are among the most widely used pesticides in agriculture. Physiologically, these thiono compounds are relatively inactive toward AChE; they must undergo oxidative desulfuration, often mediated by the cytochrome P450 liver enzyme, to convert the P=S group into the highly reactive P=O (oxon), which then binds to the enzyme [56].
3. Dithiophosphate: Characterised by the presence of two sulfur atoms associated with the phosphorus centre (typically one P=S and one P-S linkage), this group includes compounds such as malathion. The dual-sulfur configuration influences their lipid solubility and degradation pathways, often resulting in distinct environmental persistence profiles compared to simple phosphates.

Figure 1 summarises the chemical classification of OPPs based on their structural configurations [56].

The phosphorus-containing functional groups form the basis for OPP reactivity and metabolic pathways, but their specific molecular architectures, including hydrocarbon chain configuration, halogen substitution, and lipophilicity (log *K*_ow_), dictate their toxicokinetic behaviour, environmental persistence, and susceptibility to matrix interference [57]. For example, the log *K*_ow_ value reflects a compound’s tendency to partition between aqueous and organic phases and is widely used as a hydrophobicity indicator. Consequently, OPPs with different log *K*_ow_ values exhibit distinct environmental mobilities, bioaccumulation potentials, interactions with complex sample matrices, and extraction behaviours, all of which directly influence their analytical detection. OPPs constitute a chemically heterogeneous class and require tailored analytical strategies for reliable detection across diverse matrices.

### 2.2. Common OPPs of Environmental Concern

Not all OPPs are globally prevalent; rather, a subset of these compounds that possess applicability, stability, and toxicological potency have drawn regulatory and public health scrutiny [56,58]. The representative compounds presented herein were chosen for their frequent detection in environmental monitoring programmes and their established potency as AChE inhibitors [59,60]. These compounds are primarily utilised in intensive agricultural sectors to manage a broad spectrum of sucking and chewing pests [61,62]. Their physical properties determine their environmental residence time [57,63]. For example, lipophilic compounds have significant potential for bioaccumulation in aquatic food chains, whereas volatile organophosphates pose challenges related to atmospheric transport and human inhalation exposure [64]. OPP residues are persistently detected in commercial food commodities, including fresh produce and cereal grains, underscoring the need for analytical methodologies that meet the strict maximum residue limits established by international food safety authorities. Table 1 classifies these compounds based on their properties and key environmental and toxicological concerns, providing a comprehensive reference for the analytical challenges linked to their detection in complex matrices.

As summarised above, OPPs share the fundamental AChE inhibition mechanism, but such properties as their molecular weight, volatility, and environmental persistence vary significantly. This diversity requires a versatile sensing approach that can adjust to different matrix effects and residue levels in complex environmental and food samples. OPP environmental ubiquity and physicochemical diversity determine their distribution across agricultural and aquatic compartments; however, their specific molecular interactions define their risk profile. The toxicological significance of these compounds lies in their ability to perturb the cholinergic system, which is the most important mechanism in both environmental toxicity assessment and biosensing interface design. To elucidate the crucial relationship between molecular structure and biological impact, the following section examines the biochemical toxicity mechanisms, focusing on acetylcholinesterase inhibition and the subsequent physiological cascades that necessitate the development of sensitive and real-time diagnostic tools.

### 2.3. Mechanisms of Toxicity

**Figure 2:**
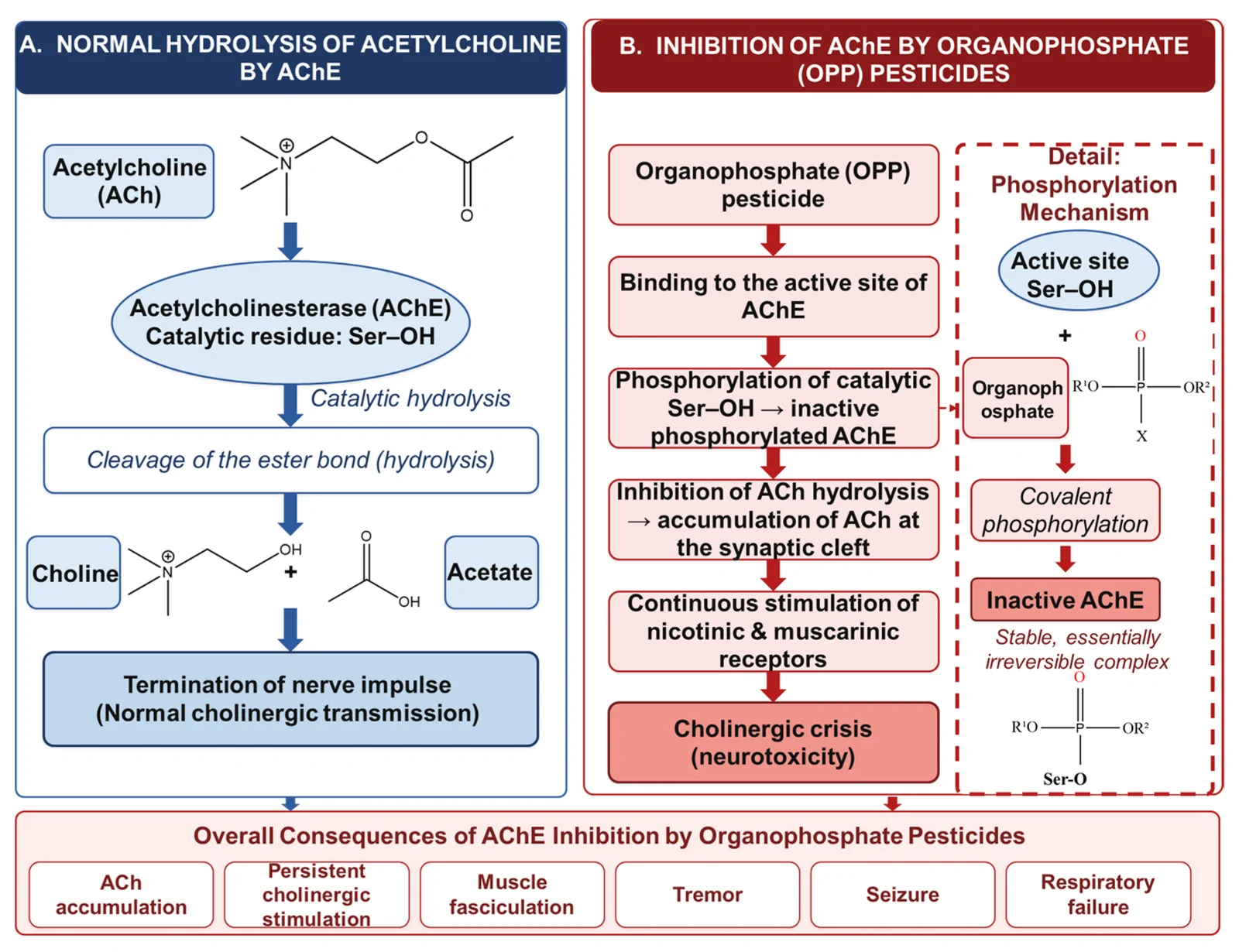
Comparison between the normal hydrolysis of acetylcholine by acetylcholinesterase (AChE) and the inhibition mechanism induced by OPPs. Panel (**A**) illustrates physiological acetylcholine hydrolysis into choline and acetate. Panel (**B**) illustrates the catalytic serine residue phosphorylation by OPPs, resulting in irreversible AChE inhibition, acetylcholine accumulation, continuous cholinergic stimulation, and neurotoxicity.

OPPs inhibit the AChE enzyme, a serine hydrolase essential for terminating cholinergic signalling [77,78]. Figure 2 schematically contrasts the normal catalytic hydrolysis of acetylcholine by AChE (Panel A) with the inhibition mechanism induced by OPPs (Panel B), highlighting the catalytic serine residue phosphorylation and the resulting enzyme inhibition. Under physiological conditions, AChE rapidly hydrolyses the neurotransmitter acetylcholine (ACh) into choline and acetate at the synaptic cleft [16]. OPPs disrupt this process by functioning as suicide substrates. The pesticide phosphorus atom undergoes nucleophilic attack by the serine residue hydroxyl group located within the enzyme active site [16]. This covalent phosphonylation creates a stable, phosphorylated enzyme complex that can no longer hydrolyse ACh [12,77] (Figure 2). ACh accumulates at the synaptic junction, leading to continuous overstimulation of the nicotinic and muscarinic receptors. The result is the characteristic cholinergic crisis, with symptoms that range from fasciculations, tremors, and seizures to respiratory failure [12,78,79]. The severity and reversibility of this inhibition depend heavily on the chemical structure of the phosphorus-containing group; some phosphorylated complexes can be ‘aged’ or reactivated, whereas others remain permanently bound, requiring the organism to synthesise new enzyme molecules [80,81].

In addition, emerging evidence indicates that chronic sub-lethal OPP exposure triggers a cascade of harmful physiological events beyond the cholinergic system [48,82]. These events include oxidative stress induction via depletion of cellular antioxidants, disruption of mitochondrial function, and activation of inflammatory signalling pathways through cytokine release [15,48], all long-term cellular perturbations. In short, OPPs are not only neurotoxins but also potential drivers of neurodegenerative, endocrine, and immunological pathologies [12,48].

Understanding OPP neurotoxicological risks requires knowledge of the biochemical pathways underlying ACh inhibition. These pathways also offer a precise mechanism for analytical detection [29]. The sensitivity of the ACh active sites to phosphorylation enables highly specific biosensing platform development in which the degree of enzyme inhibition is directly proportional to the pesticide concentration [29]. The following section discusses the electrochemical detection of organophosphate pesticide residues. We examined how electrochemical transducers, ranging from simple glassy carbon electrodes to sophisticated nanomaterial-modified interfaces, capture and amplify these biological responses, offering the prospect of a rapid, portable, and cost-effective alternative to conventional chromatographic methods.

## 3. Electrochemical Detection of OPP Residues

Recent advances in electrochemical sensing have significantly improved OPP detection [29,31], potentially offering a practical alternative to conventional chromatographic, colourimetric, and fluorescence-based analytical methods. The core of this technology is the electrochemical transducer, an interfacial component that serves as a bridge between chemical analytes and digital measurements [83]. By modulating the electrode interface with advanced materials, these sensors convert chemical recognition events, such as enzymatic inhibition or direct catalytic oxidation, into quantifiable electronic signals [31,84]. These analogue signals are then captured and processed by data acquisition systems, which digitise the input for sophisticated computational analysis, potentially enabling rapid, high-sensitivity and portable diagnostics in complex environmental matrices [29,85].

Functionally, electrochemical sensors rely on oxidation and reduction reactions that occur at the interface of a three-electrode system comprising a working, reference, and counter electrode [83]. Upon interaction with the working electrode, the analyte’s chemical constitution drives electron transfer, generating a signal that is amplified by the transducer [83]. To enhance selectivity, the electrode surface is often functionalised with specialised recognition elements, such as immobilised enzymes or molecularly imprinted polymers [86,87]. Based on these elements, sensors are broadly categorised as enzymatic or non-enzymatic [31,84]. Enzymes, such as AChE, provide exceptional specificity; however, high costs, complex purification, and sensitivity to environmental conditions often limit their utility [29,31]. Non-enzymatic platforms have emerged as alternatives that overcome these biological limitations [31,84]. Their respective advantages, limitations, and suitable application scenarios are discussed below.

Enzymatic sensors, particularly those using AChE, are highly sensitive and selective because their signal generation is directly linked to enzyme inhibition [29,31]. However, their performance is often limited, as discussed in the last paragraph [29,31]. In contrast, non-enzymatic sensors based on CPCs, carbon nanomaterials, metal nanoparticles, metal oxides, and molecularly imprinted polymers offer greater stability, lower costs, and greater suitability for portable, long-term environmental monitoring [31,84]. However, because molecular recognition in non-enzymatic sensors relies on surface interactions or electrocatalytic activity rather than specific biological binding, they may have lower selectivity and often require advanced electrode engineering or surface functionalisation to lessen interference from similar compounds and complex samples [31,84]. Therefore, the choice between enzymatic and non-enzymatic sensors depends on the intended application, considering sensitivity, selectivity, operational stability, fabrication complexity, and practical deployment requirements.

The efficacy of these sensors also depends on their transduction mode, which dictates how the signal is measured. Voltammetric methods (including cyclic, differential pulse, and square wave voltammetry) have become the primary modes for detecting OPPs. Unlike steady-state amperometry, voltammetry modulates the electrode potential over a defined range [83], which allows the sensor to distinguish specific OPP redox signals from the background noise of complex samples [88,89], providing superior peak resolution and sensitivity in many cases. Through the synergy of advanced materials and precision voltammetric measurement, electrochemical sensors provide a potentially reliable platform for pesticide residue determination [31,84].

The properties of a specific electrode interface strongly influence its performance, rendering rational material design crucial. The overall sensor performance, that is, its stability, electron transfer rate, and surface area, is dictated by the electrode interface architecture [39]. Traditional electrode materials often lack the synergistic properties required to achieve high conductivity and specific catalytic activity simultaneously [90]. To overcome these limitations, the field has increasingly pivoted toward developing CPCs [91]. These advanced materials combine the intrinsic electrical conductivity and processability of polymers with the unique electrocatalytic and structural properties of nano-reinforcements [39,91]. The following section outlines the fundamentals of CPCs, exploring how the integration of CP matrices with nanomaterials improves electron transfer, catalytic activity, and interfacial stability and supports the development of robust, field-deployable sensing platforms [31,39].

## 4. Fundamentals of CPCs

CPs have become essential components in electrochemical sensor design owing to their unique combination of electrical, mechanical, and optical properties. Materials such as polyaniline (PANI), polypyrrole (PPy), polythiophenes (PTh), and poly (3,4-ethylenedioxythiophene) (PEDOT) are particularly valuable for their high conductivity, environmental stability, and low cost [92]. In addition, these polymers offer high structural tunability, enabling facile incorporation of nanomaterials and recognition elements to create synergistic sensing interfaces.

**Figure 3:**
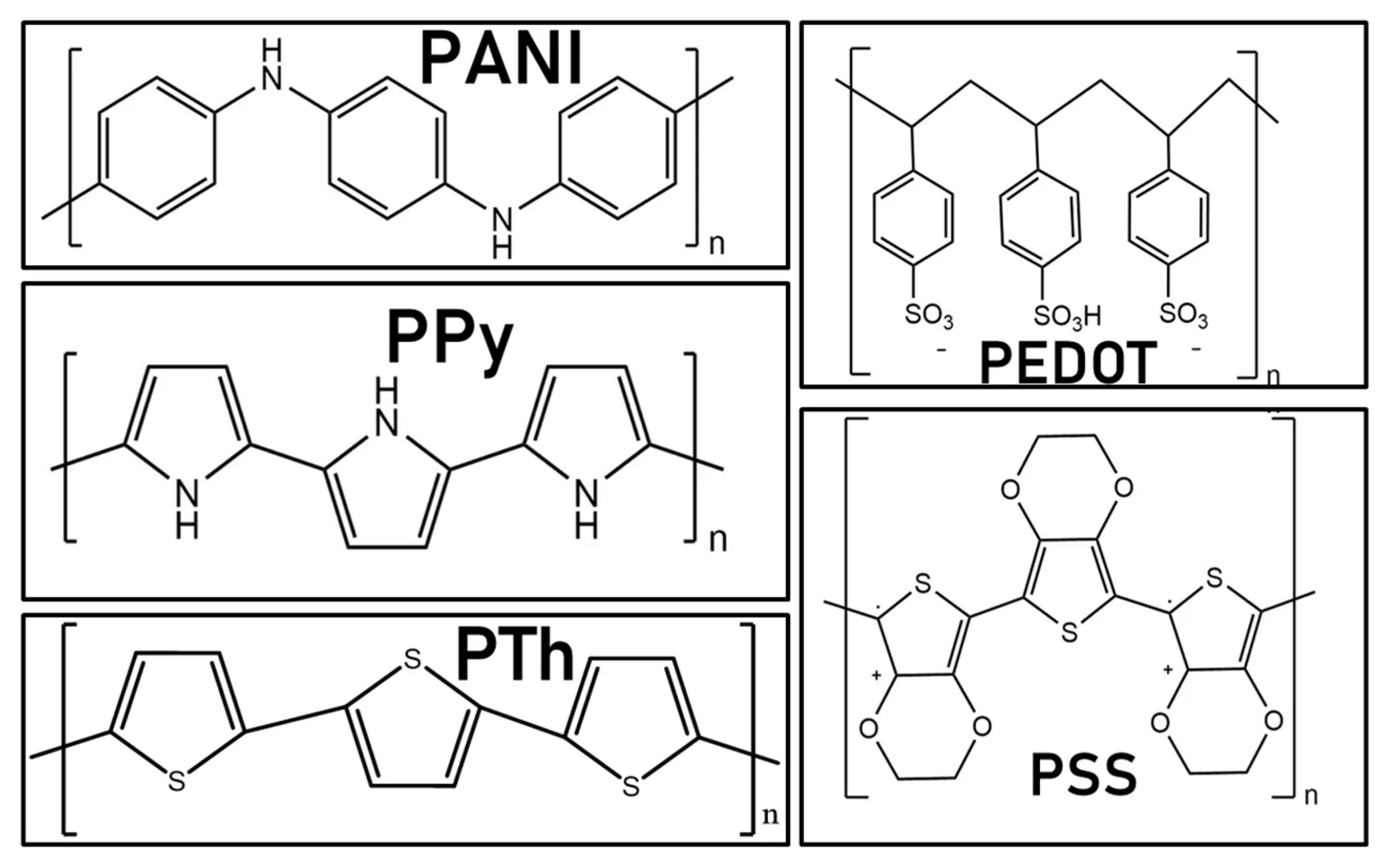
The chemical structures of the conductive polymers (CPs) commonly used in electrochemical sensors for OPP detection.

The utility of CPs is increased by their versatile deposition methods, which include drop-casting [93], chemical synthesis, and electropolymerisation [94,95], the latter of which allows control of film thickness and morphology directly on the electrode surface. The most extensively investigated OPP detection platforms to date are PANI, PPy, and PEDOT derivatives [39,89,96], which facilitate rapid electron-transfer kinetics [39], can be chemically doped [95], and are inherently compatible with the diverse analytes in agricultural matrices [97]. The chemical structures of these representative CPs are illustrated in Figure 3, and their specific modulations of sensor performance for OP detection are detailed in subsequent sections. To facilitate comparison among the CPs, Table 2 summarises their key physicochemical properties and electrochemical characteristics relevant to organophosphate sensing.

As summarised in Table 2, the four CPs differ less in their overall conductivity range than in their structural factors governing charge transport, functionalisation capability, and electrochemical behaviour. These differences determine their suitability for specific sensing strategies and nanocomposite architectures; therefore, the choice of polymer is closely related to the intended sensing application. For example, PEDOT achieves high conductivity through its highly conjugated backbone, whereas PANI depends strongly on doping state and environmental pH. The remarkable performance of CPC-based sensors arises from synergistic interaction between the CP matrix and incorporated nano-fillers, such as metal nanoparticles, carbon nanotubes, or graphene [105]. Three primary mechanisms govern this synergy. First, the high nano-reinforcement surface-to-volume ratio significantly increases the active surface area of the electrode, providing greater site density for pesticide interaction [106]. Second, the polymer backbone facilitates rapid charge transport via its conjugated π-electron system, while the embedded nanoparticles act as ‘electrochemical wires’ that bridge polymer chains, thereby reducing charge-transfer resistance across the interface [42,107]. Third, these composites often display enhanced molecular recognition; for instance, the polymer matrix can be tuned to enhance specific organophosphate molecule adsorption via π–π stacking or electrostatic interactions. Meanwhile, electrocatalytically active nanoparticles such as Au, Pt, Pd, and CuO facilitate electron transfer and provide catalytic sites, thereby reducing the overpotential required for pesticide oxidation or reduction and enhancing signal intensity and analytical sensitivity. In contrast, magnetic nanoparticles such as Fe_3_O_4_ primarily increase the electroactive surface area, improve analyte preconcentration, and enhance the structural stability of the composite rather than directly lowering the overpotential [108].

Having discussed the synergistic mechanisms and fundamental advantages of CP composites, we next examined the specific polymers that serve as the backbone for these advanced sensing interfaces. The CP selection is determined by its electron band structure, chemical stability, and processability, all of which define the sensitivity and selectivity of the sensor toward OPP residues.

The following sections provide a detailed evaluation of the most prominent CP systems, including polyaniline (PANI), polypyrrole (PPy), and the highly stable poly(3,4-ethylenedioxythiophene) (PEDOT and its derivatives, such as PEDOT:PSS) [39,43]. We also examine the structural significance of polythiophene and various specialised heterocyclic polymers that have demonstrated exceptional facilitation of rapid electron transfer [43,107]. By comparing the electrochemical profiles and environmental compatibility of these systems, we can better understand how their chemical architectures are leveraged to optimise trace-level pesticide detection in complex samples [39,97].

### 4.1. Polyaniline (PANI)

Polyaniline (PANI) is among the most studied electrochemical sensing CPs, favoured for its exceptional environmental stability, cost-effectiveness, and ease of synthesis. Beyond its robust mechanical and optical properties, PANI is attractive for its high thermal stability and flexible fabrication. It can be synthesised via chemical or electrochemical routes in diverse media for various sensing applications [99]. The electrochemical behaviour of PANI is intrinsically linked to its three primary oxidation states: leucoemeraldine (reduced), emeraldine (mixed-state), and pernigraniline (oxidised). Its reversible proton-doping mechanism enables tunable redox behaviour and integration with nanomaterials for electrochemical sensing. Its conductivity arises from delocalisation of charge carriers along the conjugated polymer backbone, a process that can be modulated to optimise charge transport and mobility [98]. Compared with PPy and PEDOT, PANI behaviour is more strongly influenced by protonation–deprotonation equilibria, which enables greater electrochemical tunability but also may reduce performance stability under neutral or alkaline conditions.

Recent advancements have leveraged PANI as a versatile structural scaffold for highly selective pesticide detection, including the development of PANI-based molecularly imprinted polymers (MIPs). For example, Liang et al. [109] developed functionalised PANI-MIPs on the surface of a glassy carbon electrode (GCE) to achieve high selectivity for parathion. The PANI-MIPs were derived from the functionalisation of PANI with CH_2_=CHCOCl to obtain FUN-PANI, followed by electropolymerisation of parathion as a template molecule to obtain FUN-PANI-MIPS. The resulting sensor achieved a remarkable LOD of 0.011 µM over a parathion concentration range of 0.034–18.67 µM. Furthermore, the developed sensor exhibited superior discrimination against structurally similar pesticides, such as parathion-methyl, paraoxon, imidacloprid, malathion, diuron, propanil, diazinon, and methiocarb.

The functional diversity of PANI allows it to serve as a robust platform for tethering catalytic complexes via ‘click chemistry’. Ipek et al. [74] pioneered the use of azide-functionalised PANI (PANI-N3) to modify GCE surfaces. A cobalt phthalocyanine bearing alkyne terminal substituents (TA-CoPc) was electrochemically bonded to the azide groups of poly(4-azido aniline) (ANI-N3) to obtain GCE/PANI-N3/TA-CoPc. This architecture facilitated rapid electron transfer, enabling sensitive parathion detection with an LOD of 1 × 10^−7^ mol L^−1^ in the concentration range 3.50 × 10^−8^–1.89 × 10^−6^ mol L^−1^. Subsequent research [110] utilised manganese phthalocyanine (TA-MnPc) on an indium tin oxide (ITO) substrate. In this work, the conductive PANI film served as an effective platform for immobilising TA-MnPc while facilitating electron transfer to form an ITO/PANI-N_3_/TA-MnPc-based sensor. The resulting sensor exhibited good analytical performance for fenitrothion and diazinon, with LODs of 0.015 μmol dm^−3^ and 0.89 μmol dm^−3^, respectively. These studies collectively underscore the versatility of PANI as both a transducer and immobilisation matrix.

### 4.2. Polypyrrole (PPy)

Polypyrrole (PPy), distinguished by its unique electronic structure, is a cornerstone of CP research. Unlike PANI, whose conductivity changes drastically with pH-dependent doping, PPy conducts through a comparatively stable oxidised state and is less sensitive to ambient conditions. The electrochemical performance of PPy is strongly influenced by its polymer morphology and chain organisation, which govern its electron-transfer efficiency and electroactive site accessibility. The PPy-based sensor performance is intrinsically tied to its morphology. The synthetic procedures used directly dictate PPy particle size, surface area, and electrical conductivity, ultimately determining the sensitivity of the final sensing interface [100].

The favourable electrical conductivity, biocompatibility, and ease of electropolymerisation of PPy render it an effective platform for immobilising and stabilising biological recognition elements [39,101]. A classic application is the immobilisation of AChE, in which PPy simultaneously stabilises the immobilised enzyme and facilitates electrochemical oxidation of the AChE-catalysed reaction product thiocholine. For instance, Dutta et al. [111] used a PPy matrix reinforced with gelatin and glutaraldehyde to trap AChE, achieving an impressive LOD of 0.12 ppb for paraoxon. This approach demonstrates that PPy can lower oxidation potentials and enhance signal transduction, bridging the gap between biological recognition and electronic output.

Integrating PPy with metallic nanomaterials further amplifies these sensing capabilities through synergistic effects. Gong et al. [112] demonstrated this by creating a three-dimensional porous network of Au nanoparticles (AuNPs) embedded within PPy nanowires. This composite architecture provided a biocompatible environment that preserved enzyme bioactivity, while the combination of AuNPs and PPyNWs significantly increased the oxidation of the enzyme’s thiocholine product, thus increasing detection sensitivity. The OP detection criteria were determined based on the AChE enzymatic activity inhibition, with an LOD of 2 ng mL^−1^ over the methyl parathion concentration range 0.005–4.5 g mL^−1^. Tefera et al. [113] combined a PPy structural scaffold with cobalt nanoparticles (CoNPs) to detect phoxim. In this configuration, the PPy chains interacted with the CoNPs as donors, significantly reducing charge-transfer resistance and increasing the electroactive surface area compared to single-component electrodes. The resulting sensor based on CoNPs/PPy/GCE demonstrated a linear relationship between the reduced peak current and the phoxim concentration over the range 0.025–12 nM, with an LOD of 4.5 nM.

Finally, the utility of PPy extends to molecular imprinting strategies, which prioritise extreme sensitivity and selectivity. Nagabooshanam et al. [114] employed a PPy matrix as a conductive support and molecular template on a gold microelectrode for chlorpyrifos detection. Utilising differential pulse voltammetry, the sensor achieved an exceptional LOD of 0.93 fM, demonstrating the potential of PPy-based molecular imprinting for detecting trace residues in complex agricultural matrices, such as fruit and vegetable juices. These diverse applications confirm that PPy is a dynamic and tunable material that can facilitate complex electrochemical interactions for high-performance pesticide diagnostics. Collectively, these studies demonstrate that PPy offers substantial versatility, functioning as an integrated sensing matrix that supports enzymatic, molecularly imprinted, and nanocomposite-based electrochemical platforms. Compared with PANI, PPy provides greater flexibility for biomolecule immobilisation, whereas compared with PEDOT, its mild electropolymerisation conditions make it particularly attractive for enzyme-based and molecularly imprinted sensing interfaces.

### 4.3. PEDOT and PEDOT:PSS

Poly(3,4-ethylenedioxythiophene) (PEDOT) is one of the most electrically conductive polymers, reaching 6259 S cm^−1^ in thin films and roughly 8800 S cm^−1^ in single crystals, only an order of magnitude below traditional metallic conductors [103]. Despite its superior conductivity, pristine PEDOT is limited by its insolubility in both water and common organic solvents, significantly restricting its practical application in solution-based fabrication. To overcome this limitation, PEDOT is frequently synthesised in the presence of poly(4-styrenesulfonate) (PSS), a water-soluble polyelectrolyte that serves as a dopant and stabiliser. The resulting dispersion, PEDOT:PSS, features a complex morphology, in which positively charged, conjugated PEDOT chains are encapsulated by the negatively charged, insulating PSS matrix [102].

Compared with PANI and PPy, PEDOT:PSS offers high electrical conductivity, excellent solution processability, and environmental stability, rendering it particularly attractive for flexible, printable electrochemical sensors. These characteristics originate from its unique phase-separated microstructure, in which conductive PEDOT-rich domains are surrounded by an insulating PSS matrix that governs charge transport, film formation, and electrical conductivity [115]. Although the PSS phase enhances aqueous dispersibility and facilitates large-area film fabrication, its insulating nature may partially limit charge transport, often necessitating post-treatment or composite engineering to maximise electrochemical performance [116]. Despite this issue, PEDOT:PSS distinguishes itself from other CPs by providing an effective balance between electrical performance, processability, and compatibility with flexible electrochemical sensing interfaces.

These characteristics have been successfully translated into electrochemical sensing applications. Hryniewicz et al. [116] demonstrated the utility of this material by fabricating a PEDOT:PSS-modified ITO electrode for the detection of paraoxon. Using linear sweep voltammetry, their sensor achieved a sensitivity of 0.1767 µA L mol^−1^ and an LOD of 4.95 µmol L^−1^. Although this detection limit is higher than those reported for several PANI- and PPy-based nanocomposite sensors discussed above, the study highlights a different strength of PEDOT:PSS. Rather than maximising analytical sensitivity, PEDOT:PSS provides excellent processability, uniform film formation, and compatibility with flexible electrode fabrication, rendering it an attractive platform for printable and large-area electrochemical sensing devices.

### 4.4. Polythiophene (PTh) and Its Derivatives

Polythiophene (PTh) and its derivatives represent an important class of CPs for electrochemical sensing because of their π-conjugated backbones, good environmental stability, and efficient charge-transport properties. Compared with PANI and PPy, PTh-based materials have been investigated less extensively but have attracted increasing interest owing to their tunable molecular structures and favourable optoelectronic characteristics. The thiophene backbone can be readily modified via side-chain functionalisation or copolymerisation, enabling control of its physicochemical properties, including hydrophobicity, electrical conductivity, and molecular recognition. These structural modifications facilitate the rational design of sensing interfaces with improved compatibility with functional nanomaterials and enhanced performance in complex environmental matrices. In contrast to PEDOT, which is usually chosen for high electrical conductivity, PTh derivatives are more frequently explored because their structures can be engineered to optimise optoelectronic properties and interfacial recognition. Furthermore, the inherent chemical stability of PTh renders it attractive for applications requiring prolonged operational durability, particularly in photoelectrochemical and hybrid sensing platforms.

The versatility of the PTh family is evidenced by the diverse performance of its derivatives, such as poly(3-hexylthiophene) (P3HT) and poly(thiophene-3-acetic acid). These materials serve as effective platforms for both electrochemical and photoelectrochemical sensing. By integrating PTh with advanced nanomaterials, such as TiO_2_ nanoparticles, graphene, or carbon nanotubes, the intrinsic electron-transfer limitations of the polymer can be bypassed. These composites create a synergistic environment where the PTh matrix acts as a conductive bridge, while the nanomaterial reinforcements provide high surface area catalytic sites, significantly lowering OPP detection limits [117,118,119]. Representative PTh-based sensing platforms have been evaluated in standard laboratory solutions and diverse real-sample matrices, including tap water, cucumber, green vegetables, parsley leaves, and biological samples such as human blood, serum, and urine [117,120,121,122]. These studies demonstrate the practical applicability of PTh-based composites for OPP detection, although broader validation across more complex environmental and food matrices remains necessary before widespread field implementation.

Furthermore, PTh is particularly well-suited for molecular imprinting technologies. The rigid yet tunable conjugated backbone can be engineered to stabilise ‘molecular recognition cavities,’ which function as selective traps for specific organophosphate molecules. This structural stability ensures that the binding sites remain intact during sensing, preventing loss of selectivity over time. The strong optical and electronic signatures of PTh derivatives enable their application in multimodal platforms, including transistor-based sensors and optical diagnostics. The integration of metal oxides and carbon-based nanomaterials into PTh matrices optimises the charge-transfer resistance and improves the operational longevity of the sensor. These combined benefits position PTh-based composites as a promising platform for the next generation of portable, high-sensitivity pesticide monitoring technologies [120,121].

### 4.5. Other CPs

Specialised materials, such as poly(indole-5-carboxylic acid) (PinCOOH), have also emerged as electrochemical interface engineering candidates. PinCOOH is particularly favoured for enzyme bioconjugation due to its streamlined polymerisation process, high chemical and thermal stability, and superior intrinsic conductivity. Unlike the more extensively studied PANI, PPy, and PEDOT systems, PinCOOH provides abundant carboxyl groups that facilitate covalent enzyme immobilisation while maintaining favourable electrochemical activity. PinCOOH films also exhibit enhanced redox activity, which is crucial for maintaining high signal-to-noise ratios in trace-level detection.

Chauhan et al. [123] effectively demonstrated the analytical potential of this material by developing an AChE-based biosensor for malathion and chlorpyrifos. By incorporating ZnS nanoparticles (ZnS-NPs) into the PinCOOH matrix, the researchers created a high surface area environment that enhanced enzyme stability and facilitated rapid electron transfer. When electrodeposited onto a gold electrode, the composite lowered the required working potential to a modest +0.2 V vs. Ag/AgCl, minimising interference from the sample matrix. This dual-material approach resulted in exceptional analytical performance, achieving nanomolar detection limits (0.1 nM for malathion, 1.5 nM for chlopyrifos) and broad linear ranges (0.1–50 nM for malathion, 1.5–40 nM for chlopyrifos). This work highlights the synergy between PinCOOH’s conductive backbone and the catalytic properties of the nanoparticles.

Another notable advancement involves polyzincon, a polymer derived from 2-carboxy-2′-hydroxy-5′-sulfoformazylbenzene. Historically recognised for its ability to extract metal ions selectively, polyzincon has been repurposed as an effective electrode modifier. Ensafi et al. [124] utilised electropolymerised polyzincon on a GCE to detect fenithrothion in real-world environmental and agricultural samples. The unique polymer chemical structure exerted a synergistic effect on fenithrothion redox behaviour, significantly enhancing the resolution of oxidation and reduction peaks. This polyzincon/GCE detector demonstrated a wide linear range (5–8600 nmol L^−1^) and a low LOD of 1.5 nmol L^−1^, validating its utility in complex food-based matrices.

Although these materials demonstrate the expanding utility of polymer-based sensing, the field remains dynamic. Several other CPs with potential utility, including polyacetylene, polyparaphenylene (PPPh), polyparaphenylene vinylene (PPV), and polyazulene, have yet to be fully exploited as electrode-modifying materials for OPP detection. These materials possess intrinsic electrical and optical properties that could be useful in sensing technologies, suggesting opportunities for future exploration, particularly in multimodal sensor development and advanced molecularly imprinted interfaces.

Although CPs provide the electrochemical framework, their analytical performance is often improved through integration with carbon-based fillers. Rather than serving only as conductive additives, carbon nanomaterials contribute distinct functions. The following section compares the major carbon-based fillers employed in CP composites for electrochemical pesticide sensing.

### 4.6. Carbon-Based Conductive Fillers

Carbon-based nanomaterials are the most widely used conductive fillers in CP composites because they provide functions complementary to those of CPs alone. These materials increase electroactive surface area, facilitate charge transfer, reduce polymer aggregation, and provide additional sites for catalyst or biomolecule immobilisation. Consequently, carbon fillers improve the overall analytical performance of CP composite sensors while complementing CP molecular recognition capabilities.

The fundamental advantage of carbon-based fillers is their inherent electronic properties. Materials such as graphene and carbon nanotubes (CNTs) possess delocalised π systems that facilitate rapid charge transport, acting as low-resistance conduits that connect the sensor interface to the underlying electrode [125]. This accelerated electron transfer is crucial for efficient transduction of electrochemical signals, especially when the contaminants occur at trace levels, as is usual for OPPs. Furthermore, the high surface-to-volume ratio of these materials, often coupled with a porous architecture, exponentially increases the density of active sites available for molecular interactions [126]. By creating an extended sensing interface, carbon fillers magnify the electrochemical response of even dilute analyte concentrations, a limitation in many traditional planar electrode designs.

The surface chemistry of these nanomaterials is highly adaptable. Through purposeful chemical functionalisation, the interface can be tailored to enhance its affinity for specific organophosphate molecules or to improve the sensor surface biocompatibility. Among the diverse array of carbon-based fillers, graphene oxide (GO), reduced graphene oxide (rGO), and carbon nanotubes (CNTs) have garnered the most intensive investigation. These materials share a fundamental carbon framework, but they differ significantly in structural morphology, surface oxygen content, and electron-transfer kinetics, each offering distinct design advantages for specific pesticide-sensing platforms. Table 3 compares the principal physicochemical characteristics and electrochemical functions of representative carbon-based conductive fillers, highlighting the trade-offs among conductivity, stability, functionalisation capability, and electron-transfer behaviour that influence their suitability for different sensing applications.

**Figure 4:**
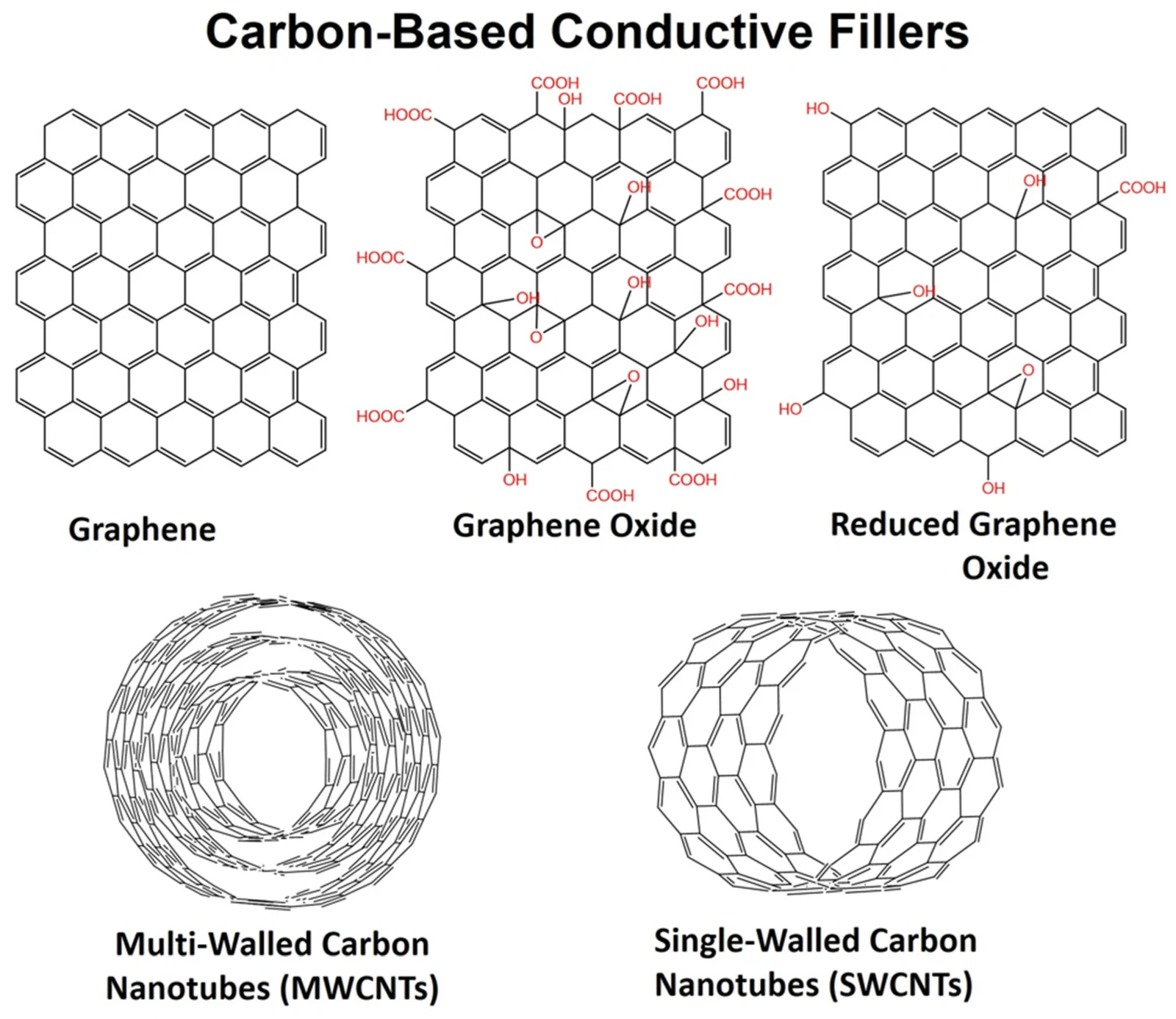
Chemical structures of graphene, graphene oxide (GO), reduced graphene oxide (rGO), single-walled carbon nanotubes (SWCNTs), and multi-walled carbon nanotubes (MWCNTs).

The structural diversity of these materials is illustrated in Figure 4, which depicts their increasing complexity from the planar sheets of graphene to the tubular geometry of nanotubes and the disordered, oxygen-functionalised structure of GO/rGO. The following sections will examine these materials in detail, analyse their structural characteristics and physicochemical properties, and summarise emerging strategies for their incorporation into high-performance electrochemical sensors that quantify OPP levels in agricultural and environmental samples. Selecting an appropriate carbon filler depends on maximising electrical conductivity and on trade-offs among surface functionalisation, structural stability, and compatibility with the intended molecular recognition strategy.

#### 4.6.1. Graphene

Graphene, the building block of all graphitic materials, is characterised by a single-layered, two-dimensional honeycomb lattice of *sp*^2^-hybridised carbon atoms. Its extraordinary performance in electrochemical sensing stems from its exceptional physical properties: it exhibits ultra-high electrical conductivity (approximately 6000 S m^−1^) and remarkable thermal conductivity (up to 5000 W m^−1^ K^−1^) [129]. Graphene’s unique electronic properties are defined by its status as a zero-gap semimetal in which both electrons and holes are mobile charge carriers. This high carrier mobility is aided by the surplus of delocalised π electrons, which are free to move across the 2D plane through the robust carbon–carbon bonds [130].

In electrochemical pesticide detection, graphene is valued for its high surface-to-volume ratio and its strong π–π stacking interactions with the aromatic rings commonly found in OPPs. Recent research has harnessed these attributes to create hybrid nanocomposites. For instance, Aghaie et al. [131] engineered NiFe bimetallic phosphosulfide/graphene nanocomposites to detect paraoxon ethyl. The graphene component served as a scaffold that promoted pesticide molecule stacking, while the bimetallic phosphosulfide provided the necessary electrocatalytic sites to boost the voltammetry signal, achieving a detection limit of 3.7 nM in the paraoxon ethyl concentration range of 0.012–10 μM.

The versatility of graphene extends to biosensing architectures. Bao et al. [132] utilised a three-dimensional (3D) graphene network integrated with copper oxide nanoflowers (3DG-CuO NPs) to support AChE for malathion detection. This 3D architecture maximised the effective surface area for enzyme loading and created a biocompatible microenvironment that preserved enzymatic activity, resulting in an ultrasensitive detection limit of 0.92 pM. Similarly, structural modifications to graphene can tailor its affinity for specific analytes; Wu et al. [100] employed β-cyclodextrin-functionalised graphene (β-CD-graphene) as an absorbent for methyl parathion. The inclusion of β-CD leveraged host-guest chemistry alongside graphene’s inherent π-electron system, enabling fast analyte accumulation and rapid electron transfer. The fabricated sensor exhibited ultra-high sensitivity for methyl parathion detection in the ranges 0.3–1.0 ppb and 1.0–500 ppb, with 0.05 ppb as the LOD.

Furthermore, the integration of graphene with biocompatible polymers and metallic nanoparticles has enabled rapid and eco-friendly sensor fabrication. Yang et al. [133] utilised an efficient electrodeposition method to create a graphene–chitosan (GR-CS) composite, combining the conductivity of graphene with the biocompatibility of chitosan to create an integrated solid-phase extraction platform. The developed sensor achieved an LOD of 0.8 ng mL^−1^ within the linear range 4.0–400 ng mL^−1^ for OPP detection. Functionalisation with metallic nanostructures has also proven effective. Singh et al. [134] developed a multifunctional platform using gold nanoparticles (AuNPs) and neutral red on biofunctionalised graphene. In this system, the platform effectively reduced the nitro groups of methyl parathion, demonstrating how rational functionalisation of the graphene surface using bovine serum albumin and electroactive dyes can be tuned to optimise sensitivity, selectivity, and operational stability in real-world environmental samples. The developed sensor exhibited linear detection ranges of 0.02–0.153 μM and 0.153–1.36 μM, with an LOD of 6 nM.

#### 4.6.2. GO & rGO

GO is a graphene derivative characterised by a structural backbone that mimics the graphite lattice, but with a crucial distinction: its basal plane and edges are heavily decorated with oxygen-containing functional groups, including hydroxyl (–OH), carboxyl (–COOH), and epoxy groups. This decoration disrupts the pristine *sp*^2^ conjugation by introducing *sp*^3^-hybridised carbon centres into the matrix [103], a unique chemical composition that causes GO to be exceptionally soluble in water and various organic solvents, which renders it highly processable. Furthermore, these oxygen groups serve as versatile ‘anchors’ for chemical functionalisation, allowing grafting of specific molecules to tailor the sensor interface for particular analytes [127]. GO is typically produced through aggressive graphite oxidation via established pathways, such as the Hummers, Staudenmaier, Brodie, and Tour methods [128].

Despite its processability, the oxygen functional-group abundance in GO significantly reduces its electrical conductivity by disrupting the delocalised π-electron network. To restore these electronic properties, GO is often reduced using chemical reagents (e.g., hydrazine) or thermal, microwave, electrochemical, photo-, or solvothermal reduction, yielding reduced GO (rGO) [135]. This reduction process eliminates a significant portion of the oxygen-containing groups, effectively restoring the conjugated π-system and significantly enhancing the electrical conductivity [136]. However, this restoration comes at the cost of water solubility, requiring the use of dispersing agents or composite strategies to maintain a stable film on the electrode surface. Although GO and rGO originate from the same parent material, they serve different functions in electrochemical sensing. GO is preferred for applications requiring extensive surface functionalisation and biomolecule immobilisation because of its abundant oxygen-containing functional groups. In contrast, rGO is more suitable for applications requiring high electrical conductivity and rapid electron transfer after partial restoration of the conjugated carbon network.

The synergistic potential of these materials is well illustrated in recent pesticide-sensing research. Yola et al. [137] exploited the high surface area of GO by integrating it with boron nitride quantum dots (BNQDs). This BNQDs/GO/GCE composite demonstrated exceptional stability and sensitivity for the detection of methyl parathion, diazinon, and chlorpyrifos. The high active-site density allowed BNQDs/GO/GCEs to detect pesticides at extremely low concentrations (down to the femtomolar range, 1.0 × 10^−12^–1.0 × 10^−8^ M), with LODs of 3.1 × 10^−13^ M, 6.7 × 10^−14^ M, and 3.3 × 10^−14^ M for methyl parathion, diazinon, and chlorpyrifos, respectively.

rGO has become a preferred scaffold for biosensors that require high electron-transfer kinetics. Velusamy et al. [138] demonstrated that rGO could be supported on cellulose microfibers (CMF) to create a hybrid CMF/rGO/SPCE interface. This composite facilitated the detection of fenithrothion at lower operating potentials, significantly broadening the linear dynamic range (0.03–1333.8 µM) and lowering the detection limit to 8 nM compared to pristine carbon surfaces. Furthermore, the high conductivity of rGO is essential for enzymatic biosensing, where rapid signal propagation is necessary to monitor pesticide hydrolysis products. Razib et al. [139] utilised rGO as a substrate for immobilising mutant phosphotriesterase (PTE) to detect paraoxon. By employing N-hydroxysuccinimide and N-(3-dimethylaminopropyl)-N′-ethylcarbodiimide (EDC) as cross-linkers to anchor the enzyme to the rGO surface, the researchers achieved enhanced electron transfer between the enzymatic byproduct (*p*-nitrophenol) and the electrode. These studies collectively confirm that although GO is valued for its functionalisation versatility and processability, rGO remains the premier choice for applications where maximising electrical conductivity and electron-transfer speed is paramount.

#### 4.6.3. Carbon Nanotubes: Single-Walled (SWCNT) & Multi-Walled (MWCNT)

Carbon nanotubes (CNTs) represent a transformative class of nanomaterials characterised by their cylindrical nanostructures, which are composed of rolled-up sheets of *sp*^2^ hybridised carbon atoms. These tubes typically feature diameters in the nanometre range, while their lengths can extend into the micron range [125]. Synthesised primarily through techniques such as chemical vapour deposition, arc discharge, and laser ablation, CNTs are broadly categorised into two types: single-walled carbon nanotubes (SWCNTs), consisting of a single graphene cylinder, and multi-walled carbon nanotubes (MWCNTs), which comprise multiple concentric graphene cylinders. Compared with graphene-based fillers, carbon nanotubes reinforce composite structures while more effectively preventing nanosheet restacking. Consequently, they are particularly suitable for CP composites that require efficient charge transport together with good mechanical stability.

CNTs have become indispensable in electrochemical sensor fabrication and consistently outperform conventional carbon materials because of their unique combination of properties: an exceptionally large specific surface area that enhances sensitivity, robust mechanical and chemical stability, and inherent biocompatibility for enzyme immobilisation. Furthermore, both the open ends and sidewalls of these nanotubes can be functionalised, enabling the creation of multifunctional sensing platforms. Owing to their high aspect ratio and superior electron transport kinetics, they are ideal for creating ultrasensitive nanoarrays and facilitating rapid signal transduction [140].

Applications of CNTs in OPP detection often focus on their ability to facilitate rapid adsorption and promote efficient charge transfer. Musameh et al. [141] utilised CNT ‘web-modified’ electrodes as a binder-free architecture to achieve rapid detection of methyl parathion. The porous, interlaced nature of the CNT network provided high adsorption capacity and minimised signal interference, achieving a detection limit of 1 nM in the range 20–1000 nM. Beyond simple adsorption, CNTs are often incorporated into complex nanocomposites to enhance catalytic activity. Putra et al. [142] developed a non-enzymatic sensor based on a hydroxylated MWCNT/graphene composite for the detection of paraoxon ethyl. In this system, the nanotubes served a dual role: they functioned as ‘electrochemical wires’ that bridged the graphene sheets to improve overall electron-transfer efficiency, while their hydroxyl groups provided sites for catalytic interaction. This synergistic hybridisation resulted in a highly sensitive platform with a linear detection range of 0.1–100 μM and a detection limit of 10 nM.

The architecture of CNTs also allows them to be used as structural spacers. Aris et al. [143] reported an innovative Ti_3_C_2_T_x_/MWCNT-OH nanocomposite sensor. A critical challenge in Ti_3_C_2_T_x_-based sensing is the tendency of their 2D sheets to restack, which drastically reduces their active surface area. By incorporating MWCNT-OH as conductive spacers, the authors successfully prevented restacking while creating efficient, high-speed pathways for charge transport. The resulting sensor exhibited a linear range of 0.1–100 μM, a detection limit of 10 nM, and a sensitivity of 0.957 μA μM^−1^ cm^−2^ while maintaining high selectivity and sensitivity in complex agricultural matrices, such as fruit samples. These studies demonstrate that CNT utility in electrochemical sensing extends far beyond simple conductivity. By acting as structural scaffolds, spacers, and efficient electrocatalytic centres, SWCNTs and MWCNTs remain at the forefront of CPC technology. They provide the necessary stability and signal-to-noise ratio improvements required for the next generation of portable, field-deployable pesticide detection tools.

### 4.7. Comparative Evaluation of CPs and Carbon-Based Fillers

CPs differ less in their overall conductivity range than in the structural factors that govern their electrical performance, and these differences largely determine their suitability for specific electrochemical sensing strategies. Among the CPs discussed in this review, PEDOT generally exhibits the highest electrical conductivity. Unlike PANI, whose conductivity is strongly influenced by protonation, the electrical performance of PEDOT is governed primarily by the structural organisation of its conjugated backbone, although controlling this structural disorder remains an active area of research [103]. However, this superior electrical performance is accompanied by reduced processability because pristine PEDOT is poorly soluble in common solvents. Consequently, PEDOT is commonly combined with poly(styrene sulfonate) (PSS) to enable solution processing and deposition onto flexible substrates, thereby expanding its applicability in printable and wearable electrochemical sensors [102].

In contrast, PANI exhibits electrical conductivity that is highly dependent on its protonation state and the surrounding pH. This characteristic reduces conductivity stability under certain operating conditions, but it also provides abundant functional groups for chemical modification, rendering PANI particularly suitable for molecular imprinting and surface functionalisation strategies, including the PANI-based MIP and click-chemistry sensors discussed in Section 4.1 [98,99].

PPy exhibits intermediate characteristics between PANI and PEDOT. Its electrical performance is strongly influenced by polymerisation conditions, dopant selection, and film morphology [100]. Consequently, the analytical performance of PPy-based sensors depends on the intrinsic properties of the polymer and the fabrication parameters that regulate the surface morphology and electron-transfer efficiency. Compared with these materials, PTh has been explored less extensively for electrochemical pesticide sensing. Nevertheless, its good environmental stability and resistance to oxidative degradation make it particularly attractive for applications requiring prolonged operational durability, especially in photoelectrochemical sensing platforms.

These structural characteristics directly influence the choice of molecular recognition strategy. Enzymatic biosensors generally favour PPy or PEDOT use because the relatively stable PPy oxidised state and the biocompatible PEDOT:PSS matrix provide more favourable environments for enzyme immobilisation than does PANI’s pH-dependent doping behaviour [111,144]. In contrast, MIP-based sensors benefit from the chemical functionalisation capability of PANI and the structural rigidity of PTh, both of which contribute to maintaining stable imprinted cavities during repeated binding and regeneration cycles [109,120]. PEDOT and PEDOT:PSS are particularly attractive for direct electrocatalytic and flexible sensing platforms because their combination of high electrical conductivity and excellent processability facilitates rapid electron transfer without requiring biological recognition elements [145].

Carbon-based fillers exhibit a similar balance between electrical performance and surface functionality. In compacted form, graphene, multi-walled carbon nanotubes (MWCNTs), and carbon black display broadly comparable electrical conductivity, with packing density and particle contact influencing the measured conductivity nearly as much as the intrinsic properties of the material itself [125]. Despite its excellent conductivity, pristine graphene possesses relatively few surface functional groups, limiting direct chemical functionalisation on the basal plane [126]. Oxidation to GO introduces abundant hydroxyl, epoxy, and carboxyl groups that improve aqueous dispersibility and facilitate chemical modification. However, disruption of the conjugated carbon network simultaneously reduces electron-transfer efficiency [127]. Partial reduction of GO restores electrical conductivity by removing oxygen-containing functional groups; however, this process also decreases the number of available active sites for subsequent functionalisation. Consequently, rGO represents a compromise between electrical conductivity and surface functionality rather than combining the maximum advantages of both GO and graphene [128]. Unlike graphene-based materials, CNTs provide one-dimensional conductive pathways that facilitate electron transport throughout the composite while simultaneously reinforcing its structural integrity, allowing the CNTs to function as conductive bridges between different components within the sensing interface.

These complementary characteristics explain why the sensing platforms discussed in Section 4.6.1, Section 4.6.2 and Section 4.6.3 employ different carbon-based fillers depending on the intended sensing mechanism. Graphene is generally preferred when rapid electron transfer and π–π interactions are required, particularly for analytes containing aromatic structures. GO is commonly selected when extensive chemical functionalisation or immobilisation of recognition elements is required because of its abundant oxygen-containing functional groups. In contrast, rGO is often employed when improved electrical conductivity is prioritised after sufficient functionalisation has been achieved. CNTs are primarily incorporated as structural reinforcements that improve electrical connectivity, minimise nanosheet restacking, and promote the formation of conductive three-dimensional networks within composite electrodes.

Overall, no single CP or carbon-based filler consistently outperforms all others across every analytical criterion summarised in Table 2 and Table 3. Successful CPC design relies on selecting materials whose physicochemical properties complement the intended recognition mechanism rather than identifying a universally superior material. For CPs, this balance typically involves electrical conductivity, environmental stability, and chemical functionalisation capability, whereas carbon-based fillers require optimisation between electron-transfer efficiency, surface chemistry, and structural reinforcement. Consequently, the rational integration of CPs with carbon-based fillers, rather than selection of any individual material, ultimately determines sensor sensitivity, selectivity, operational stability, and suitability for practical electrochemical sensing applications. These structure–property relationships provide the basis for the sensing mechanisms discussed in Section 5.

## 5. CPC-Based Electrochemical Sensors: Detection Mechanisms

Building upon the structure–property relationships discussed in Section 4, conductive polymer composites (CPCs) support electrochemical sensing through diverse molecular recognition mechanisms rather than through the conductive polymer alone. CPC-based electrochemical sensors can be classified into enzymatic inhibition, molecularly imprinted polymer-based recognition, and direct electrocatalytic sensing. Each strategy offers advantages and limitations with respect to selectivity, operational stability, fabrication complexity, and suitability for practical environmental monitoring [97,146,147].

### 5.1. Enzymatic Inhibition-Based Detection

Enzymatic electrochemical biosensors possess exceptional selectivity, specifically leveraging the interaction between OP compounds and cholinesterase enzymes, such as acetylcholinesterase (AChE) or butyrylcholinesterase (BChE). In these biosensors, OPPs function as potent inhibitors; they bind to the enzyme’s active-site serine residue via irreversible phosphorylation and suppress the enzymatic hydrolysis of substrates such as acetylthiocholine. Since the enzymatic products are electrochemically active, this inhibition results in a measurable decrease in the oxidation current, allowing for indirect, yet highly sensitive, quantification of pesticide concentration [148].

The efficacy of these sensors relies heavily on the immobilisation matrix design. Conductive polymers provide an ideal microenvironment for this purpose, offering porous, conductive, and biocompatible networks that stabilise the enzyme while maintaining rapid signal propagation [39,148]. For example, Dutta and Puzari [111] used PPy stabilised by gelatin and glutaraldehyde to effectively entrap AChE, creating a robust interface for thiocholine oxidation. Similarly, Gong et al. [112] employed a 3D porous network of Au nanoparticle-modified nanowires. This architecture not only preserved enzymatic bioactivity but also created a “nano-amplification” effect, significantly increasing the detection sensitivity for methyl parathion.

Recent studies have focused on the hybridisation of conductive polymers with carbon-based fillers and metal oxides to further optimise their performance. Kaur et al. [144] utilised PEDOT-functionalised MWCNT to create a superior redox microenvironment for AChE, facilitating the detection of chlorpyrifos-methyl. Furthermore, the integration of metal oxides, such as cerium oxide (CeO_2_), has proven to allow sensitive detection of chlorpyrifos-methyl. Several recent studies have also incorporated metal oxides into conductive polymer matrices to improve catalytic activity and enzyme immobilisation. Paneru et al. [96] successfully demonstrated that CeO_2_/PANI and CeO_2_/PEDOT:PSS composites could significantly enhance catalytic activity and sensor conductivity, even when deposited on a flexible substrate such as conductive paper. Finally, 3D architecture design, such as the graphene-CuO nanoflower platform developed by Bao et al. [132], has lowered detection limits into the ultra-trace (picomolar) range by optimising both enzyme loading and mass transport.

Compared with molecularly imprinted polymer-based and direct electrocatalytic sensors, enzymatic platforms generally provide the highest molecular selectivity because signal generation originates from specific enzyme–substrate interactions. However, their practical application remains constrained by irreversible enzyme inhibition, limited storage stability, sensitivity to environmental conditions such as pH and temperature, and the cost and complexity associated with enzyme purification and immobilisation. Consequently, recent research has increasingly focused on non-enzymatic sensing strategies that offer greater operational robustness, longer service life, and improved suitability for continuous environmental monitoring, while avoiding the intrinsic limitations of biological recognition elements.

### 5.2. Non-Enzymatic Detection Based on Molecularly Imprinted Polymers (MIPs)

MIPs, often referred to as ‘artificial antibodies’, represent a robust, synthetic alternative to biological receptors. They are synthesised by polymerising functional monomers around a target template molecule; once the template is removed, the resulting polymer matrix retains precisely engineered cavities that complement the analyte in shape, size, and chemical functionality. Because of their superior chemical and thermal stability, cost-effectiveness, and long shelf life, MIPs have become the gold standard for non-enzymatic recognition in complex environmental matrices.

CPs are particularly suitable matrices for molecular imprinting because they provide structural support for imprint formation and efficient electrochemical signal transduction simultaneously. Compared with enzymatic biosensors, MIP-based sensors eliminate biological instability while providing excellent chemical robustness and long-term storage stability. Despite these advantages, however, incomplete template removal, template leakage, and cross-reactivity toward structurally similar compounds remain important limitations affecting their analytical reproducibility and selectivity.

Liang et al. (2022) demonstrated the efficacy of this approach by functionalising PANI to improve the affinity between the conductive matrix and parathion template molecules, resulting in high selectivity over structurally similar organophosphates [109]. Similarly, Nagabooshanam et al. [114] utilised PPy as a single imprinting material on gold microelectrodes for chlorpyrifos detection, proving that a single conductive matrix can provide both structural integrity for the cavities and the electrical pathways required for DPV analysis.

To extend the performance of ultra-trace detection, recent research has focused on hybrid conductive nanocomposites. By integrating carbon-based fillers, such as MWCNTs and graphene, into the MIP matrix, researchers can significantly enhance the analytical performance of the sensor. Anirudhan et al. [89] developed a surface-imprinted copolymer of thiophene acetic acid and EDOT, reinforced with MWCNTs. In this system, the MWCNTs functioned as a “conductive highway,” accelerating electron-transfer kinetics and ensuring that even deeply embedded recognition cavities remained electrochemically accessible. Furthermore, metallic oxide or graphene inclusions can function as structural support, preventing the imprinted cavity collapse that is a common failure mode in traditional MIPs.

Recent studies further demonstrate the continued development of MIP-based electrochemical platforms toward improved selectivity and practical applicability. Saad et al. [NEW] employed computationally guided screening of functional monomers followed by electropolymerisation and Au nanoparticle deposition to develop a chlorpyrifos sensor, demonstrating how molecular-level interaction modelling can be incorporated into MIP design. The resulting sensor was successfully applied to chlorpyrifos determination in pesticide products and water samples. Similarly, Huang et al. [149] developed a surface-imprinted polymer sensor incorporating carboxylated multi-walled carbon nanotubes and graphene oxide for chlorpyrifos detection, achieving a detection limit of 1.85 × 10^−11^ mol L^−1^ with good repeatability, stability, and selectivity. Zhong et al. [150] further demonstrated a MIP/Cu-MOF/rGO/AuNPs/GCE architecture for sensitive detection of electroneutral organophosphorus pesticide residues, highlighting the continuing trend toward multifunctional recognition and signal-amplification interfaces. These recent developments indicate that current MIP research is increasingly focused not only on lowering detection limits but also on rational recognition-site design, hybrid nanostructuring, and applicability to complex samples.

### 5.3. Direct Electrocatalytic Oxidation- and Reduction-Based Detection

Direct electrocatalytic sensing offers a powerful, label-free alternative to enzymatic or imprinted platforms for the quantification of organophosphate (OPP) detection. Unlike methods that rely on biological recognition or structural cavities, this approach utilises the inherent electroactivity of the pesticide molecules themselves. Many organophosphate compounds feature redox-active functional groups, such as nitro groups (–NO_2_) or phosphorus-bearing moieties, that undergo discrete electrochemical reduction or oxidation at a highly active electrode surface. CPCs serve as ideal transducers, providing robust conductive support and actively lowering the overpotential required for these redox reactions to occur.

**Figure 5:**
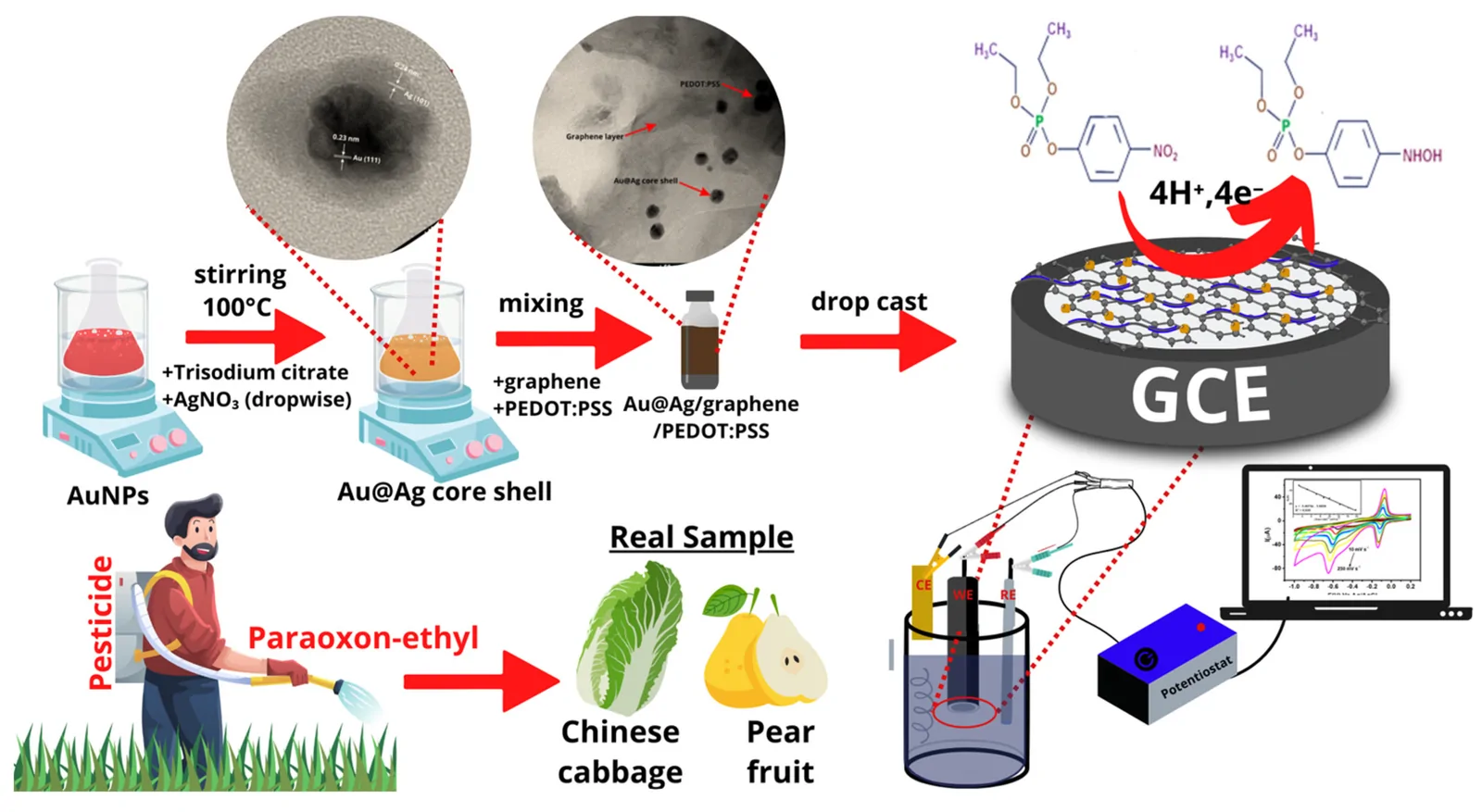
Schematic illustration of the fabrication workflow and electrochemical sensing strategy of an Au-Ag core–shell nanoparticle/graphene/PEDOT:PSS composite-modified glassy carbon electrode (GCE) for paraoxon-ethyl detection. Au-Ag core–shell nanoparticles are synthesised, integrated with graphene and PEDOT:PSS, and deposited onto a GCE to form a multifunctional sensing interface for sensitive electrochemical detection of paraoxon-ethyl in agricultural samples. Reproduced from Wahyuni et al. [145] under the Creative Commons Attribution (CC BY) licence.

The performance of these sensors is fundamentally determined by the ‘synergistic interface’ engineered at the electrode surface. By integrating CPs with advanced nanomaterials, researchers can create surfaces that drastically reduce charge-transfer resistance while simultaneously providing high-density catalytic sites. As illustrated in Figure 5, the fabrication of a multifunctional PEDOT:PSS sensing platform involves the sequential synthesis of Au-Ag core–shell nanoparticles, their integration with graphene and PEDOT:PSS, and their subsequent deposition onto a glassy carbon electrode to construct a hierarchical electrochemical interface. This architecture enables intimate contact among the functional components, maximising electrical connectivity and catalytic efficiency. Wahyuni et al. [145] demonstrated the power of architectural hybridisation by developing a graphene/PEDOT:PSS composite modified with Au-Ag core–shell nanoparticles. In this design, the core–shell nanoparticles functioned as localised catalytic “hotspots,” where plasmonic effects and enhanced electron-transfer pathways facilitated the sensitive reduction of paraoxon-ethyl. The PEDOT:PSS matrix ensured that these metallic centres remained well-dispersed and electrically connected, resulting in significant amplification.

Furthermore, the integration of two-dimensional (2D) materials, such as MXenes, has opened new avenues for low-potential sensing. Hasnan et al. [151] reported a complex composite consisting of PEDOT:PSS, MXene, bovine serum albumin (BSA), and graphene oxide (GO) for the detection of chlorpyrifos. The inclusion of MXenes was pivotal; their high metallic conductivity and hydrophilic surfaces enabled the sensor to operate at significantly reduced potentials, a crucial advantage, as operating at lower potentials effectively minimises the background noise generated by common interferences in environmental samples.

A particularly recent development is the use of carbon nanotube-coated NH_2_-UiO-66 metal–organic frameworks for direct and universal electrochemical detection of organophosphorus pesticides. Zhang et al. [152] demonstrated that the CNTs@NH_2_-UiO-66 interface can combine the intrinsic electroactivity and pesticide-binding capability of the MOF with the enhanced conductivity provided by carbon nanotubes, thereby enabling direct detection without enzyme inhibition or cascade catalytic reactions. Using paraoxon as a model analyte, the sensor achieved a linear range of 0.01–100 ng mL^−1^ and a detection limit of 2.71 pg mL^−1^. Importantly, the sensing mechanism was further supported by theoretical analysis, illustrating a recent shift toward mechanistically guided and recognition-assisted direct electrochemical sensing of OPPs.

Compared with enzymatic and MIP-based platforms, direct electrocatalytic CPC sensors offer practical advantages: they are significantly more robust, free from biological denaturation constraints, and considerably cheaper to fabricate. These sensors are simpler in design, avoiding the complex immobilisation protocols required for enzymes or the template-extraction steps needed for MIPs. However, this simplicity presents a unique challenge: selectivity. Because the detection mechanism relies on the analyte’s generic redox activity, structurally similar electroactive compounds in complex matrices (e.g., soil, wastewater) can produce overlapping signals. Consequently, the future of this field lies in “fine-tuning” the electrocatalytic interface using specific modifiers to ensure that only the target pesticide molecule can oxidise or reduce at the desired potential, thereby improving analytical reliability and facilitating practical environmental monitoring.

Enzymatic, molecularly imprinted polymer-based, and direct electrocatalytic sensors each demonstrate excellent analytical performance for OPP detection; however, their relative strengths and limitations become more apparent when evaluated collectively. Therefore, the following section provides a comparative analysis of these three CPC-based recognition strategies to facilitate selection of the most appropriate sensing approach for different analytical applications.

### 5.4. Comparative Analysis of CPC-Based Recognition Strategies

The three CPC-based sensing mechanisms discussed in the previous sections exhibit complementary analytical characteristics rather than a universal performance hierarchy. Their differences stem from fundamentally distinct recognition mechanisms, leading to different balances among selectivity, operational stability, fabrication complexity, and practical applicability. Consequently, selecting an appropriate sensing strategy depends on the analytical objective and the intended operating environment rather than maximising a single performance parameter, such as analytical sensitivity. To facilitate direct comparison, Table 4 summarises the principal characteristics of the enzymatic inhibition, MIP-based recognition, and direct electrocatalytic sensing strategies.

Enzymatic biosensors achieve the highest molecular selectivity because signal generation originates from highly specific interactions between OPPs and the AChE active site. This biological recognition minimises interference from non-target compounds and enables sensitive detection even at trace concentrations. However, this same recognition mechanism also represents the principal enzymatic platform limitation. Enzyme activity is inherently affected by environmental conditions, including pH and temperature, while irreversible inhibition progressively decreases the catalytic activity over repeated uses. Consequently, enzyme purification, immobilisation, storage, and preservation of biological activity increase fabrication complexity and reduce long-term operational stability, serious drawbacks despite the excellent analytical sensitivity of these sensors [96,154].

MIP-based sensors provide an intermediate approach by replacing biological recognition with synthetic recognition cavities that complement the target pesticide. This strategy largely overcomes the biological instability associated with enzymatic biosensors while maintaining high selectivity toward predetermined analytes, as demonstrated by PANI-based MIP sensors for parathion and PPy-based MIP sensors for chlorpyrifos [109,114]. However, analytical performance depends strongly on the quality of the imprinted cavities. Incomplete template removal, template leakage, and cross-reactivity toward structurally related compounds may reduce analytical reproducibility and long-term selectivity [120]. Although fabrication requires template synthesis, polymerisation, and template extraction, the absence of biological handling generally provides greater operational stability than enzymatic platforms.

Direct electrocatalytic sensing follows a fundamentally different recognition mechanism, because analyte detection relies on the intrinsic pesticide electrochemical activity rather than on a biological receptor or synthetic recognition cavity. As no enzyme immobilisation or template preparation is required, direct electrocatalytic sensors generally offer the simplest fabrication procedure and excellent operational stability. Representative examples include the Au-Ag core–shell/graphene/PEDOT:PSS sensor for paraoxon-ethyl and the PEDOT:PSS-MXene-BSA-GO composite for chlorpyrifos, which demonstrate the advantages of CPCs in facilitating rapid electron transfer and straightforward sensor fabrication [145,151]. However, the absence of a highly specific recognition element inevitably reduces the selectivity because oxidation or reduction may also occur for structurally related electroactive compounds present in complex sample matrices.

Overall, these three recognition mechanisms should be regarded as complementary rather than competing approaches for OPP detection. Enzymatic biosensors remain the preferred option when maximum molecular selectivity is required under controlled laboratory conditions. MIP-based sensors provide an effective balance between selectivity and long-term operational stability, making them particularly suitable for repeated field monitoring of specific target pesticides. In contrast, direct electrocatalytic sensors prioritise simplicity, rapid response, and low fabrication cost, making them attractive for portable monitoring and preliminary screening applications where rapid decision-making is more important than compound-specific identification. Therefore, the selection of an appropriate CPC-based sensing strategy should be guided by the intended analytical application, balancing selectivity, operational stability, fabrication complexity, and practical deployment requirements rather than relying solely on analytical sensitivity.

## 6. Recent Advances in CPC-Based OPP Sensors

### 6.1. PANI-Based Composite Sensors

Polyaniline (PANI) is a cornerstone of electrochemical pesticide sensing, distinguished by its unique blend of high electrical conductivity, environmental robustness, and cost-efficient synthesis. In the context of OPP detection, PANI functions as more than a simple conductor; it serves as a versatile structural scaffold that promotes rapid electron transfer while providing a rich surface for the anchoring of functional nanomaterials, including metal oxides, noble metal nanoparticles, and carbon derivatives, as well as biological recognition elements such as enzymes or nucleic acids. Its conjugated π-electron backbone acts as an ‘electronic highway’ for charge transport, while its inherently porous morphology significantly increases the electroactive surface area, facilitating deeper analyte interaction. As summarised in Table 5, PANI-based sensors have been successfully applied using both enzymatic and non-enzymatic sensing mechanisms, achieving detection limits ranging from the micro- to picomolar level depending on the sensing architecture.

**Figure 6:**
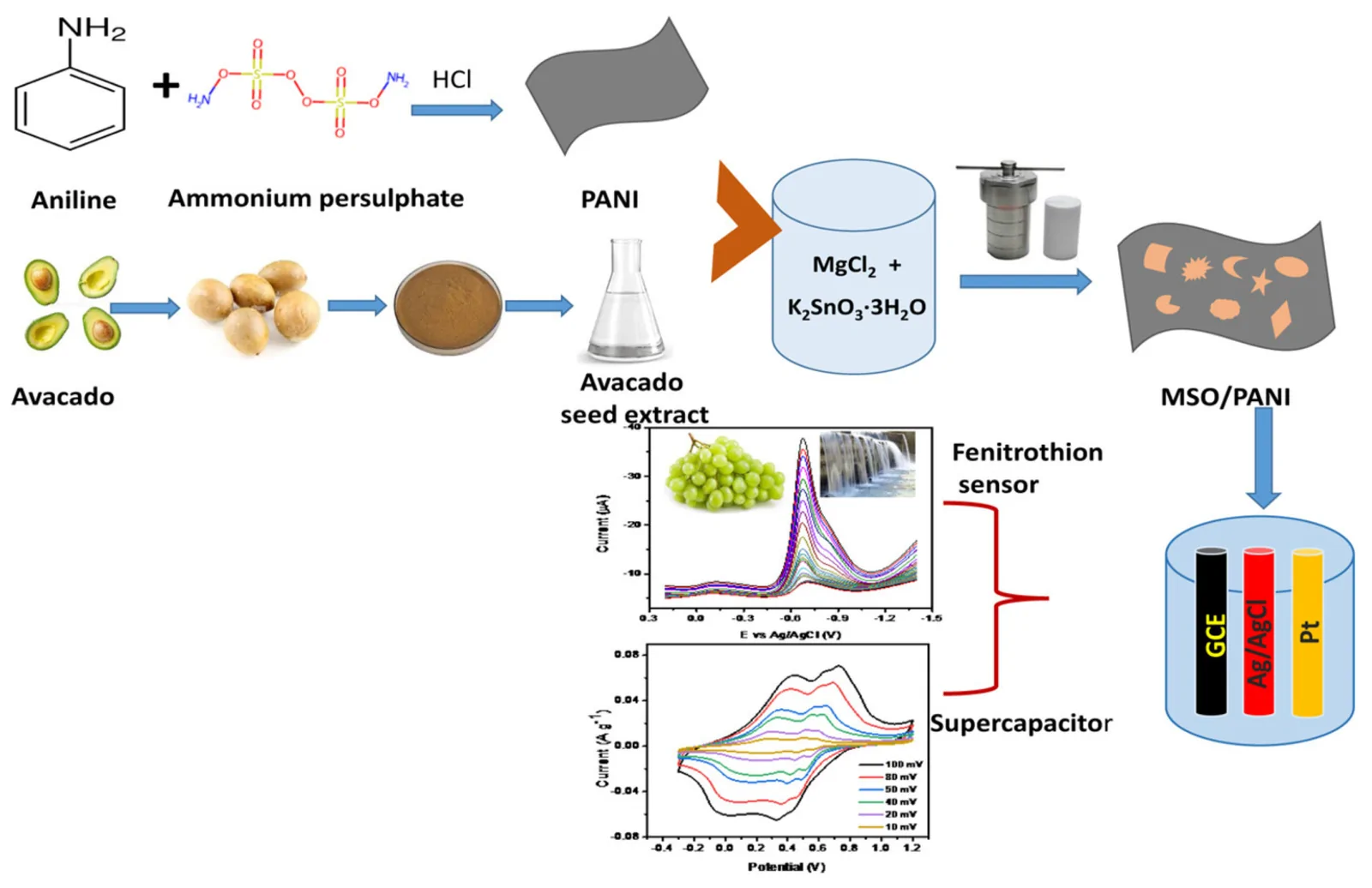
Representative fabrication strategy of a metal oxide/polyaniline (MSO/PANI) composite for electrochemical pesticide sensing. The synthesis combines green-produced Mg_2_SnO_4_ nanoparticles with conductive polyaniline to construct a nanocomposite electrode for fenitrothion detection with high electrocatalytic performance. Reproduced from Jyothi et al. [156] under the CC BY licence.

In current research, metal oxide/PANI composites often deliver the most impressive analytical metrics, largely due to a synergistic effect in which the metal oxide provides catalytically active sites and PANI enhances the overall conductivity of the transducer. For instance, Sudharsan et al. [155] developed a Cu_x_O-PANI nanocomposite-modified GCE for chlorpyrifos detection, achieving a linear range of 1–12 µM and an ultra-low LOD of 0.54 pM. Similarly, Paneru et al. [96] reported an AChE/CeO_2_@PANI/ITO biosensor for paraoxon-ethyl detection with a sensitivity of 0.59 µA (pM)^−1^ cm^−2^ and an LOD of 0.65 pM, while demonstrating satisfactory recoveries in carrot and soil samples. Comparable performance was observed for Mg_2_SnO_4_/PANI nanocomposites by Jyothi et al. [156], who reported a broad linear range of 0.01–390 µM and an LOD of 0.46 nM for fenitrothion detection. The fabrication strategy, shown in Figure 6, employed green-synthesised Mg_2_SnO_4_ nanoparticles embedded within a conductive PANI matrix to form a hybrid sensing platform that enhanced charge transfer and electrocatalytic activity. Likewise, TiO_2_-en-NC-g-PANI electrodes, developed by Sharma et al. [165], exhibited a wide detection range of 1.0 × 10^−12^–1.0 × 10^−6^ M for malathion, a sensitivity of 32.64 mV µM^−1^ cm^−2^, and long-term stability exceeding 60 d. These findings suggest that metal oxide incorporation improves conductivity and introduces catalytic and adsorption sites, thereby significantly enhancing analytical sensitivity.

Carbon nanomaterials represent another major class of PANI composite components. Incorporating carbon nanotubes, graphene derivatives, or graphene quantum dots generally improves electron mobility, electroactive surface area, and structural stability. Wang et al. [158] developed a PANI/N-GQD sensor for profenofos detection, which achieved a broad linear range of 1 × 10^−1^–1 × 10^4^ ng mL^−1^ with an LOD of 3.59 ng mL^−1^ and satisfactory recoveries in vegetable samples. Similarly, Nagabooshanam et al. [160] reported a PANi/fMWCNT/ITO sensor capable of simultaneously detecting chlorpyrifos and methyl parathion with detection limits of 8.8 and 10.2 ng L^−1^, respectively. Reduced graphene oxide (rGO) has also been successfully integrated with PANI-based composites. Thangarasu et al. [169] developed an MnO_2_/PANI/rGO nanocomposite for methyl parathion detection, which displayed a sensitivity of 3.05 µA µM^−1^ cm^−2^, a detection limit of 7.4 nM, and a rapid response time of approximately 3 s. These studies indicate that carbon nanomaterials primarily enhance charge-transport pathways and increase the accessible surface area, thereby improving sensor responsiveness and operational stability.

The incorporation of noble metal nanoparticles enhances the electrochemical performance by improving the conductivity and catalytic activity. Sun et al. [157] developed a chlorpyrifos PANI/AuNPs biosensor with an LOD of 7.90 × 10^−5^ ppm and excellent reproducibility. Wei et al. [164] fabricated a multilayer Au/PANI/MWCNT/AChE biosensor for parathion-ethyl detection using a boron-doped diamond electrode. The sensor exhibited dual linear ranges of 0.2–2 nM and 8–100 nM, with an LOD of 0.062 nM and recovery values of 90.09–99.44% in apple skin samples. The enhanced performance of noble metal-modified systems is generally attributed to accelerated electron transfer and improved immobilisation efficiency of the biological recognition components.

PANI-based OP sensors can also be broadly categorised into enzymatic and non-enzymatic platforms. Enzymatic biosensors dominate because AChE-based inhibition mechanisms provide excellent selectivity toward organophosphate compounds. Huang et al. [159] developed a three-dimensional porous AChE/AgNPs/GO/PANI/SPCE biosensor for dimethyl methylphosphonate and omethoate, achieving remarkably low detection limits of 6.43 × 10^−5^ ppb and 1.07 × 10^−6^ ppb, respectively. Somerset et al. [161] reported an early Au/MBT/PANI/AChE/PVAc biosensor capable of detecting diazinon and fenthion with detection limits below 0.2 ppb. Maanaki et al. [85] developed a MWCNT/PAnNF-based biosensor integrating AChE and BChE for biomonitoring applications, demonstrating excellent reproducibility and agreement with conventional radiometric methods. The high sensitivity observed in these systems arises from the strong amplification effect associated with enzymatic inhibition.

In contrast, non-enzymatic and alternative biorecognition systems have attracted increasing attention because they avoid enzyme denaturation, complex immobilisation procedures, and limited storage stability. Prabhakar et al. [166] developed a PANI-PVS-dsCT-DNA/ITO sensor that can detect chlorpyrifos and malathion with detection limits of 0.5 ppb and 0.01 ppm, respectively, while maintaining stability for up to six months. Similarly, Wang et al. [171] fabricated a ZrO_2_/ordered macroporous PANI electrode for methyl parathion detection with an LOD of 2.28 × 10^−10^ M, highlighting the potential of direct electrochemical sensing strategies. Mekonnen et al. [172] further demonstrated the applicability of multifunctional semiconductor-PANI composites through a PANI-CdS/CeO_2_/Ag_3_PO_4_ electrode that achieved a malathion detection limit of 3.0 × 10^−9^ M. In addition, Du et al. [163] developed a PANI-PPy-MWCNTs/AChE biosensor for malathion detection that exhibited dual linear ranges of 0.01–0.5 µg mL^−1^ and 1–25 µg mL^−1^ with a detection limit of 1.0 ng mL^−1^.

Despite these advancements, a significant gap remains between laboratory-scale precision and field-ready robustness. Enzymatic sensors provide superior sensitivity but are hindered by protein denaturation. Conversely, non-enzymatic sensors offer durability but struggle with the selectivity challenges of real-world chemical matrices. Moving forward, the field is shifting toward multifunctional hybrid architectures that combine the catalytic power of metal oxides with the robust selectivity of molecular imprinting or highly specific bioreceptors. The ultimate objective is to integrate these sophisticated PANI-based CPs into portable, low-power devices capable of autonomous and real-time pesticide quantification in environmental and agricultural settings.

### 6.2. PPy-Based Composite Sensors

Polypyrrole (PPy) has become an important CP choice for electrochemical diagnostics. Its appeal lies in its unique combination of high electrical conductivity, environmental stability, and easy electropolymerisation, enabling the deposition of thin, uniform films directly on the electrode surface. Beyond its electrical properties, the PPy backbone is rich in nitrogen-containing functional groups, which serve as excellent anchoring sites for chemical and biological functionalisation. This versatility enables PPy to serve as a porous, biocompatible matrix for many recognition elements, including enzymes, nucleic acids, metallic nanoparticles, and MIPs. As summarised in Table 6, PPy-based sensors have demonstrated excellent analytical performance for multiple OP pesticides, achieving detection limits from the micromolar range in early sensor designs to the femtomolar level in advanced molecularly imprinted and nanocomposite architectures.

The evolution of PPy-based OP sensors mirrors the broader progress in electrochemical pesticide diagnostics, which has transitioned from simple conductive coatings to high-performance, nanostructured hybrid architectures. Early research proved the concept; for instance, studies by Hailin et al. [181] demonstrated that PPy-modified electrodes could successfully resolve the electrochemical signals of various pesticides, including methyl parathion, methyl azinphos, parathion, and fenitrothion, through direct voltammetric analysis. Early biosensing work by Sulak et al. [182] pioneered AChE immobilisation within a PPy matrix, establishing the now-standard enzyme-inhibition mechanism for paraoxon detection and achieving a sensitivity of 1.77 nA μM^−1^. These early systems displayed modest analytical performance compared with contemporary sensors, but they established the foundation for the development of more sophisticated PPy-based sensing architectures.

Among sensing strategies, enzymatic biosensors remain a focus because of the exceptional selectivity provided by AChE inhibition in the presence of OP compounds. In these systems, PPy serves as a conductive matrix and biocompatible microenvironment that immobilises the enzyme while preserving its catalytic activity. Simultaneously, the polymer facilitates the crucial electron transfer between the biological recognition event and the electrode surface. Integrating conductive nanomaterials and noble metal nanoparticles into the PPy framework has significantly improved its analytical performance. For instance, Loguercio et al. [173] developed a PPy-IC-AuNP-AChE/FTO biosensor for methyl parathion detection, achieving a detection limit of 24 fmol L^−1^ over a wide concentration range. The addition of AuNPs significantly boosted electron-transfer efficiency, leading to a marked sensitivity increase compared to AuNP-free benchmarks. Likewise, Du et al. [89] demonstrated the power of hybrid architectures by developing an AuNPs/PPy/HOOC-MWCNTs/GCE sensor for methyl parathion detection. By combining the conductive properties of PPy with the catalytic advantages of AuNPs and functionalised CNTs, the authors achieved an LOD of 5.0 nM, underscoring the synergistic effects of nanostructuring on accelerating charge-transfer kinetics and elevating overall sensing performance.

The integration of advanced nanomaterials further broadened the scope of PPy-based biosensing. Wang et al. [175] pioneered an AChE-Chit/PPy/NiFe LDH/Ti_3_C_2_ MXene/GCE biosensor for dimethoate detection, achieving an exceptionally low LOD of 1.459 × 10^−11^ mol L^−1^. In this architecture, the hierarchical conductive network formed by PPy, layered double hydroxides (LDH), and MXene nanosheets accelerated electron transport and created a high active-site density for enzymatic reactions. Similarly, Pan et al. [176] utilised an MXene/CNHs/PPy/AChE/GCE biosensor for methyl parathion, reporting an ultra-wide linear range of 0.002–346 ng mL^−1^ and an ultra-low detection limit of 0.00021 ng mL^−1^. These studies underscore the strategic advantage of hybridising PPy with MXenes and carbon nanostructures: PPy acts as a physical barrier to prevent nanosheet aggregation, thereby maintaining a high effective surface area while simultaneously boosting the conductivity and enzyme immobilisation efficiency. These findings confirm that precise engineering is critical for improving the analytical performance of PPy-based enzymatic biosensors to meet the standards required for trace-level pesticide monitoring.

**Figure 7:**
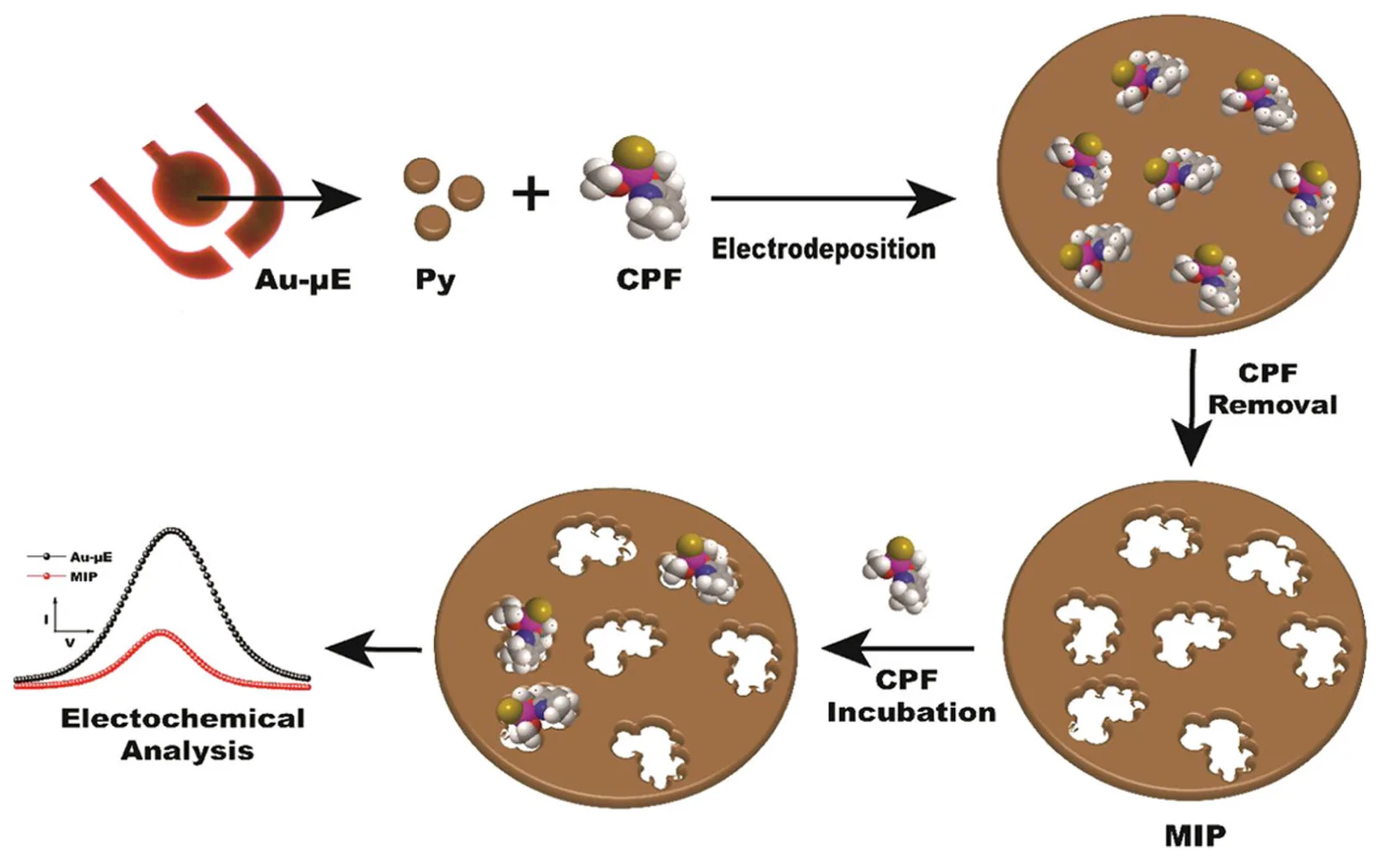
Schematic illustration of fabrication and sensing mechanism of a molecularly imprinted polypyrrole (MIP-PPy) microelectrode for chlorpyrifos (CPF) detection. Pyrrole is electropolymerised in the presence of CPF as the template molecule to form an imprinted PPy film on a gold microelectrode (Au-μE). Subsequent template removal generates selective recognition cavities, which enable CPF rebinding during electrochemical analysis. Reproduced from Nagabooshanam et al. [114] under the Creative Commons Attribution (CC BY) licence.

MIP-based PPy sensors have also become a promising OPP detection strategy. Unlike enzyme-based systems, MIP sensors employ artificial recognition sites that physically complement the target analyte, providing high selectivity without relying on biological components. As a result, MIP-PPy sensors often exhibit superior chemical stability, lower fabrication costs, and enhanced resistance to environmental fluctuations compared to enzyme-based systems. Uygun et al. [177] demonstrated the feasibility of chlorpyrifos-imprinted PPy electrodes through a label-free impedimetric sensing platform with a detection limit of 4.5 μg L^−1^. Subsequent developments significantly improved analytical performance. The general MIP-PPy sensor fabrication strategy is illustrated in Figure 7, where pyrrole is electropolymerised in the presence of chlorpyrifos to generate a molecularly imprinted polymer film. The chlorpyrifos template is then removed to create selective recognition cavities complementary to the target analyte, enabling highly selective rebinding during electrochemical detection. Nagabooshanam et al. [114] reported a MIP-PPy/Au microelectrode for chlorpyrifos detection with a remarkable working range from 1 fM to 1 μM and an ultra-low detection limit of 0.93 fM. Likewise, Radi et al. [178] developed a PPy-based molecularly imprinted sensor for profenofos detection with nanomolar sensitivity, while Capoferri et al. [183] demonstrated selective dimethoate detection using a dimethoate-imprinted PPy platform. The continued reduction in detection limits observed across these studies demonstrates that combining molecular imprinting with CP matrices can be a highly selective and sensitive pesticide detection strategy.

A unique extension of MIP-based PPy technology was reported by Capoferri et al. [180], who developed an electrochromic molecularly imprinted sensor that integrated iridium oxide (IrO_x_) nanoparticles with PPy for chlorpyrifos detection. The sensor achieved a remarkable LOD of 100 fM and maintained a broad dynamic range from 100 fM to 1 mM, illustrating that PPy can function as both a conductive sensing matrix and an active component in multifunctional signal-transduction systems. This innovation suggests that the potential applications of CP-based pesticide sensors may extend beyond conventional electrochemical detection. Beyond enzymatic and MIP-based designs, alternative recognition mechanisms using PPy composites have also emerged. Prabhakar et al. [179] developed a dsCT-DNA/PPy-PVS/ITO sensor capable of detecting chlorpyrifos and malathion via DNA-based recognition mechanisms. DNA-based sensors often exhibit higher LODs than the most advanced enzymatic or imprinted platforms, but they offer unique opportunities to study pesticide–biomolecule interactions and are a viable alternative for selective sensing. Similarly, Tefera et al. [113] demonstrated the versatility of PPy by developing a CoNPs/PPy/GCE sensor for phoxim detection with an LOD of 4.5 nM, confirming that PPy can effectively support direct electrochemical sensing approaches that operate independently of biological recognition elements.

The analytical performance metrics summarised in Table 5 highlight several key trends in PPy-based organophosphate (OP) sensor evolution. First, hybrid nanocomposite architectures consistently outperform pristine PPy films, confirming that simple conductivity enhancement alone is insufficient to achieve ultra-low detection limits. Instead, the synergistic interplay between PPy and advanced nanostructured materials, such as MXenes, carbon nanomaterials, and metal nanoparticles, is the primary driver of superior sensor performance. Second, MIP-based PPy sensors often achieve analytical sensitivities that rival or exceed those of enzymatic biosensors while providing significantly greater chemical and operational stability. Third, sensors reinforced with MXenes and metal nanoparticles consistently exhibit the broadest linear ranges and the lowest detection limits among enzymatic sensors. Finally, the dramatic progression from micromolar detection limits in early designs to femtomolar levels in contemporary systems underscores the maturation of PPy-based sensing technology.

Despite these technical milestones, obstacles to practical application remain. Enzymatic PPy biosensors are often hindered by protein denaturation, limited long-term shelf life, and susceptibility to environmental pH and temperature fluctuations. MIP-based platforms offer a more robust alternative, but they often suffer from incomplete template removal and inherent variability in cavity formation, which can compromise sensor-to-sensor reproducibility. Reliance on complex, multi-layered architectures that integrate polymers with MXenes, noble metals, and carbon-based fillers increases fabrication difficulty and potentially impedes large-scale commercial viability. Finally, the lack of standardised reporting protocols for electrolyte composition, calibration methods, and sensitivity units continues to make direct comparisons between research studies difficult [39]. Addressing these challenges through standardised fabrication and more consistent evaluation metrics will be essential for transitioning these high-performance sensors from the laboratory to real-world commercial use.

The evolution of PPy-based OPP sensors parallels a paradigm shift from simple conductive polymer films to multifunctional architectures that integrate molecular imprinting, nanostructured fillers, and specific biological recognition elements. The data summarised in Table 5 show that molecularly imprinted PPy platforms currently achieve some of the lowest reported detection limits and most robust operational stability, whereas enzymatic nanocomposite sensors remain the benchmark for high selectivity and wide analytical ranges. This distinct performance profile suggests that in the next generation of PPy sensors, these two development lines should converge, combining the structural selectivity and chemical stability of molecular imprinting with the powerful signal amplification capabilities of advanced nanocomposite frameworks. Future research should also emphasise improving reproducibility, simplifying fabrication procedures, and integrating PPy-based sensing platforms into portable and real-time monitoring devices for environmental surveillance, food safety assessment, and on-site pesticide screening.

### 6.3. PTh and Derivative-Based Composite Sensors

PTh and its derivatives constitute a vital class of intrinsically conductive polymers, valued for their superior electrical conductivity, exceptional environmental stability, and highly tunable optoelectronic properties. Their molecular structure also allows for facile structural modification, making them uniquely adaptable for specific sensing tasks. Although PTh-based composites have historically received less attention for OPP detection than PANI or PPy, research has demonstrated that they are highly effective sensing platforms. As highlighted in Table 7, PTh-based systems utilise a diverse array of strategies, including enzymatic, molecularly imprinted, and photoelectrochemical mechanisms, to achieve impressive analytical performance, with detection limits advancing from early micromolar designs to contemporary systems capable of picomolar and sub-picomolar sensitivity.

Early research in this field focused on leveraging the inherent electrochemical properties of PTh as a robust transducer material for biosensing. A landmark study by Guler et al. [121] established the viability of this approach by developing an AChE-based biosensor using poly([2,2′;5′,2″]-terthiophene-3-carbaldehyde) for malathion detection. This sensor exhibited a linear response over the concentration range of 0.015–1.644 mM with a detection limit of 4.08 nM. The aldehyde-functionalised terthiophene structure proved highly effective, providing a reliable scaffold for enzyme immobilisation and maintaining the favourable electron-transfer kinetics necessary for sensitive inhibition-based pesticide detection. This study demonstrated the suitability of functionalised thiophene derivatives and underscored the inherent versatility of PTh platforms in building sophisticated, task-specific biosensors.

Recent research has increasingly focused on optimising PTh performance through nanostructured filler integration and advanced functionalisation strategies. These hybrid designs attempt to overcome the inherent limitations of neat polymer films and test the boundaries of sensitivity and operational utility. Recent advances have sought to integrate PTh with nanostructured materials to enhance charge-transfer efficiency and analytical performance. For example, Ramachandran et al. [122] developed a MnO_2_/PTh/rGO/GCE nanocomposite for methyl parathion detection, where the synergistic combination of PTh, rGO, and MnO_2_ nanoparticles significantly enhanced the electroactive surface area and accelerated electron-transfer kinetics. This sensor exhibited a broad linear range of 0.5–65 μM and a detection limit of 5.72 nM. It demonstrated a successful application in complex biological matrices, including blood, serum, and urine. The synergistic interaction among these components enhanced electrocatalytic activity for methyl parathion oxidation. Similarly, Şen Gürsoy and Kahraman [184] reported a PTh/TiO_2_ composite electrode for malathion detection in which the incorporation of TiO_2_ nanoparticles improved the interfacial charge-transfer process.

MIP-based PTh sensors have emerged as a high-performance alternative to composite materials, often achieving superior selectivity. Anirudhan et al. [120] fabricated a molecularly imprinted poly(TAA-co-EDOT)/MWCNT composite for chlorpyrifos detection that integrated thiophene-based copolymers and MWCNTs. The platform achieved an ultra-low detection limit of 4 × 10^−12^ M within a broad linear range of 0.02–1000 nM. This exceptional performance was attributed to the combination of precise molecular recognition, enhanced conductivity, and the increased surface area provided by the MWCNT network. Such studies confirm that molecular imprinting is a powerful tool for overcoming selectivity issues frequently encountered in traditional electrochemical pesticide sensing.

Finally, PTh derivatives have shown remarkable promise in the emerging field of photoelectrochemical (PEC) sensing. Many thiophene-based polymers are semiconductors, facilitating efficient photoinduced charge separation and offering a unique signal-transduction pathway. Li et al. [117] developed a P3HT/TiO_2_/GCE photoelectrochemical sensor for chlorpyrifos detection with an LOD of 0.01 μM, whereas Xu et al. [118] reported a PEC sensor based on a polythiophene derivative film (PS2TTz) that achieved a detection limit of 0.36 μg L^−1^. The developed sensor was fabricated through a simple one-step electropolymerisation process and exhibited a wide linear range of 1–218.92 μg L^−1^ for chlorpyrifos detection. In this mechanism, interaction between the pesticide and polymer film inhibited photoinduced electron transfer, leading to a measurable decrease in photocurrent. These findings highlight the dual advantage of PTh derivatives: they enable both electrical conductivity and photoactivity to be harnessed for high-precision analytical performance.

The analytical performance metrics summarised in Table 7 show the evolution and versatility of PTh-based pesticide sensors. While PANI and PPy have historically dominated the field owing to their established protocols, the data for PTh reveal a unique capability to function effectively across a broader modality spectrum, including enzymatic biosensors, molecularly imprinted sensors, direct electrocatalysis, and photoelectrochemical (PEC) systems. The strategy of integrating PTh with advanced fillers, such as MWCNTs, rGO, and metal oxide nanoparticles, to optimise charge-transfer kinetics and maximise electroactive surface area has been successful in nearly all cases. MIP-based PTh composites have emerged as the most successful composites in achieving sub-picomolar detection limits, validating the coupling of precise molecular recognition with robust conductive matrices. Furthermore, the emergence of PEC PTh offers a compelling, distinct signal-transduction route that exploits the inherent optoelectronic properties of thiophene derivatives, providing a potential alternative to traditional electrochemical monitoring.

However, the field of PTh-based sensing is still maturing. Several challenges must be addressed for PTh to match the development level of its PANI and PPy counterparts. The literature remains limited, and multistep syntheses of complex, functionalised thiophene monomers and hybrid nanocomposites pose obstacles to scalable production. Additionally, as with all CP sensor classes, the lack of standardised calibration protocols and experimental conditions complicates the cross-comparison of analytical performance. Addressing issues of operational stability, device-to-device reproducibility, and the transition from laboratory prototypes to field-deployable units will be essential.

Ultimately, PTh-based CPCs represent a high-potential frontier in OPP detection. Their tunable electronic structures and unique photoactivity suggest that the next phase of research should move beyond simple sensor development and focus on high-level integration. Future efforts should prioritise the convergence of molecular imprinting with photoelectrochemical transduction, the simplification of fabrication pathways, and the design of portable, real-time sensing devices. By refining these multifunctional architectures, PTh-based sensors are well positioned to contribute significantly to environmental surveillance and food safety diagnostics.

### 6.4. PEDOT and PEDOT:PSS-Based Composite Sensors

Poly(3,4-ethylenedioxythiophene) (PEDOT) and its water-dispersible derivative, poly(3,4-ethylenedioxythiophene:poly(styrenesulfonate) (PEDOT:PSS), have emerged as sophisticated alternatives to PANI, PPy, and PTh. These materials are valued for their mechanical flexibility, superior electrical conductivity, and exceptional environmental stability, properties that uniquely fit them for next-generation flexible electronic sensing platforms. Their compatibility with diverse nanomaterials and biological recognition elements has enabled the development of extremely sensitive sensing architectures for multiple OPPs, including chlorpyrifos, paraoxon, and malathion. As detailed in Table 8, these architectures have progressed from early micromolar detection limits to state-of-the-art systems identifying analytes at femtomolar and sub-part-per-billion levels.

Early research into sensors based on PEDOT focused primarily on its role as a conductive transducer. Manisankar et al. [195] demonstrated the efficacy of a simple PEDOT-modified electrode for chlorpyrifos detection with an LOD of 0.29 μg L^−1^. Following this proof-of-concept, the field quickly pivoted toward enzyme-based biosensor development, where the ability of PEDOT to facilitate rapid charge transport while maintaining a biocompatible interface proved highly advantageous. Istamboulie et al. [185] then developed an AChE-based screen-printed biosensor, which achieved detection limits for chlorpyrifos-oxon detection in the low nanomolar range (4 × 10^−9^ M), effectively proving the utility of PEDOT in low-potential amperometric applications.

To extend these limits toward ultra-trace detection, modern design strategies have focused on PEDOT hybridisation with high-surface-area carbon nanomaterials, such as MWCNTs, graphene, and reduced graphene oxide (rGO). These carbon-based fillers dramatically increase the electroactive surface area and provide a scaffold for robust bioreceptor immobilisation, significantly outperforming pristine polymer films. This synergy was utilised by Kaur et al. [154], who developed a PEDOT-MWCNT/FTO bioelectrode for malathion detection. By leveraging the combined conductive pathways of PEDOT and carbon nanotubes, these researchers achieved an extraordinary detection limit of 1 fM, with a wide linear range from 1 fM to 1 μM. Similarly, PEDOT-wrapped MWCNT electrodes enabled chlorpyrifos-methyl detection at sub-ppb levels, highlighting the synergistic effect of conductive polymers and carbon nanostructures in achieving ultra-trace pesticide monitoring [144].

Current research on PEDOT sensing has shifted toward engineering multifunctional nanocomposites. Researchers are incorporating a broad spectrum of advanced materials, including MXenes, metal nanoparticles, metal oxides, and metal–organic frameworks (MOFs), to access new levels of electrocatalytic efficiency. The integration of components such as Ti_3_C_2_Tx MXene, Pd-Fe_3_O_4_ nanoparticles, and ZIF-derived structures has fundamentally altered the sensing interface, facilitated accelerated electron transfer kinetics, and provided specialised adsorption sites that have lowered detection limits into the nanomolar and picomolar ranges.

This drive for enhanced performance has also spurred a move toward portable, low-cost diagnostics, particularly through the development of flexible and paper-based PEDOT biosensors. These platforms leverage the lightweight and disposable nature of paper substrates while maintaining the high electrochemical sensitivity of PEDOT. Notable studies, such as the AChE/CuO@PEDOT paper-based biosensor reported by Paneru et al. [188], demonstrate the viability of this approach for field-deployable pesticide screening. More recently, an AChE/CuO@PEDOT paper biosensor was developed for paraoxon-ethyl detection, with CeO_2_ incorporation further improving analytical performance and lowering the detection limit to 0.12 nM [194]. Concurrently, Bohra et al. [189] further advanced these capabilities with a CuS@rGO/PEDOT platform, achieving an extraordinary detection limit of 0.28 pM for fenithrion. This system demonstrates that the strategic synergy between PEDOT and high-conductivity nanomaterials can overcome the signal attenuation issues traditionally inherent in paper-based substrates.

Next-generation device architectures have deployed PEDOT successfully, including ion-sensitive field-effect transistors (ISFETs), ‘electronic tongues’ arrays, and photoelectrochemical (PEC) devices. These platforms exploit the unique electrical and optoelectronic properties of PEDOT. A powerful example is the organic photoelectrochemical transistor developed by Yang et al. [191], which achieved an ultra-low chlorpyrifos detection limit of 2.2 fg mL^−1^. This result illustrates the immense promise of PEDOT-based PEC amplification strategies for future high-sensitivity sensing applications.

In summary, PEDOT and PEDOT:PSS are perhaps the most adaptable conductive polymer families for OPP detection. Their combination of superior conductivity, mechanical flexibility, and compatibility with diverse recognition elements has enabled a technological trajectory from simple micromolar-level sensors to femtogram-per-millilitre-resolution devices. However, the field faces hurdles around long-term stability, device-to-device reproducibility, and the complexities of large-scale manufacturing. Future research must enhance sensor robustness, integrate wireless communication for real-time data transmission, and develop intelligent, wearable sensing systems that provide continuous, on-site monitoring for food safety and environmental protection.

Moreover, the gap between high-performance research and widespread commercial implementation remains a critical hurdle. To translate the current successes in sensing sensitivity and architectural design into real-world applications, the field must address persistent technical and systemic challenges.

### 6.5. Comparative Analysis of CPC-Based Electrochemical Sensors

The previous sections reviewed recent advances in CPC-based electrochemical sensors according to individual polymer families. However, direct comparison among these sensing platforms is essential to identify the factors that govern their analytical performance and practical applicability. Because the reported studies employ different conductive polymers, composite architectures, recognition strategies, and sensing mechanisms, evaluating them collectively provides a broader understanding of how structural and physicochemical characteristics influence sensor behaviour beyond individual performances. Collective evaluation also helps to identify common design principles that enhance sensitivity, selectivity, operational stability, and successful analysis of real food and environmental samples.

To provide this overview, Table 9 summarises 20 representative CPC-based electrochemical sensors for OPP detection, highlighting their representative composite materials, target analytes, recognition strategies, electrochemical techniques, analytical performance, operational stability, real sample applications, key advantages, and current limitations. Rather than simply compiling published studies, this table enables critical comparison of current research trends and illustrates how differences in composite architecture, interfacial properties, and recognition chemistry collectively influence sensor performance.

A comparison of the representative studies summarised in Table 9 reveals several recurring trends that distinguish the highest-performing CPC-based electrochemical sensors from those with more limited analytical performance. Rather than reiterating the results presented in the table, the subsequent discussion identifies the underlying structural and physicochemical drivers of sensitivity and stability, explaining why specific composite architectures outperform others.

One of the clearest observable trends in Table 9 is that the lowest detection limits are generally achieved by composite electrodes rather than by conductive polymers used alone. In most high-performing systems, the conductive polymer is integrated with a secondary conductive, catalytic, or photoactive material that enhances electron transport and signal generation. For example, the PEDOT-based organic photoelectrochemical transistor (OPECT) incorporating a TiO_2_/CdS heterojunction achieved an exceptionally low detection limit of 2.2 fg mL^−1^, substantially lower than electrocatalytic platforms such as the MnO_2_/PANI/rGO sensor for methyl parathion (7.4 nM). This difference is consistent with the role of secondary functional materials in improving interfacial electron transfer kinetics and reducing charge-transfer resistance beyond the capability of the conjugated polymer backbone alone [201]. In the OPECT platform, the TiO_2_/CdS heterojunction functions as a photogate that modulates the PEDOT:PSS channel current, converting a subtle chelation event at the semiconductor interface into a significantly amplified electrical signal [191]. This mechanism explains the remarkable analytical sensitivity of the device despite the absence of a biological recognition element. Table 9 also shows that this platform achieved a considerably lower detection limit than the MXene/CNHs/PPy composite, which relied primarily on a single catalytic component rather than a heterojunction architecture [176]. However, because only one heterojunction-based CPC sensor is represented in Table 9, this comparison should be interpreted cautiously and considered an indication of potential rather than conclusive evidence of the superiority of heterojunction architectures.

Analytical sensitivity alone, however, does not fully reflect sensor performance. The most successful CPC sensors in Table 9 combine low detection limits with good operational stability, and these characteristics are commonly associated with porous, well-organised composite architectures rather than dense or poorly dispersed polymer films. For instance, the MXene/CNHs/PPy/AChE sensor retained 88.6% of its initial response after 15 d while maintaining a detection limit of 0.00021 ng mL^−1^. In contrast, the structurally simpler PPy-IC-AuNP-AChE sensor showed a pronounced dependence on storage conditions, retaining, after 7 d, 84% of its activity at −15 °C but only 44% at 4 °C. This difference reflects the important roles of electroactive surface area (ECSA) and composite morphology in controlling both electron transfer and mass transport within the sensing interface [202]. Although MXene nanosheets possess a high theoretical surface area, they readily restack during fabrication. Incorporating carbon nanohorns effectively suppressed this restacking, preserving accessible active sites and creating additional ion-diffusion pathways throughout the composite [176]. A similar structural optimisation is observed in the CeO_2_@PEDOT:PSS paper-based sensor, where ethylene glycol decreased the coulombic interaction between PEDOT and PSS, promoted polymer-chain reorientation, and improved integration with the cellulose substrate [194]. As a result, the sensor retained 92.6% of its activity after 25 d. Together, these examples suggest that the superior performance of the highest-ranked sensors in Table 9 is associated with optimised composite morphology, rather than with the intrinsic conductivity of the CP itself. Optimising composite morphology simultaneously increases electroactive surface area, facilitates electron transfer, and improves long-term stability. However, this interpretation is based on a limited number of well-characterised composite systems and should be validated using a broader range of CPC architectures.

The comparison presented in Table 9 also clarifies that the choice of recognition strategy involves balancing sensitivity, stability, and selectivity rather than maximising a single analytical parameter. AChE inhibition remains the most sensitive recognition approach, with detection limits reaching 0.062 nM for the Au/PANI/MWCNT platform and 0.12 nM for the CeO_2_@PEDOT:PSS sensor. However, these systems consistently exhibit limited storage stability because analytical performance depends on preserving the native conformation and catalytic activity of the immobilised enzyme [154]. In contrast, MIP-based sensors demonstrate greater chemical stability and regeneration capability. The pencil graphite electrode and microfluidic MIP platforms retained 98.7% and 88.2% of their initial responses after 30 and 70 days, respectively, indicating that synthetic recognition cavities can provide stable, reusable recognition sites while avoiding enzyme degradation, although interference from structurally related metabolites may still occur [114,177]. Aptamer-based sensors represent an intermediate strategy by combining molecular specificity with reversible target binding and regeneration [200]. However, in PANI-based sensors, the analytical performance of an aptamer-based system may still be influenced by PANI protonation requirements, which are not always compatible with optimal aptamer stability [158]. Direct electrocatalytic sensors eliminate biological recognition elements, simplifying fabrication and improving operational robustness, although they generally sacrifice molecular selectivity toward structurally similar organophosphate compounds. Overall, no single recognition strategy provides the best performance under all analytical conditions, and the most appropriate approach ultimately depends on the intended application and the balance required between sensitivity, stability, and selectivity.

Evidently, excellent analytical sensitivity under laboratory conditions does not guarantee successful performance in real samples. Instead, practical applicability appears to depend largely on the immobilisation chemistry stability and the sensing interface compatibility with complex sample matrices. Sensors employing covalent immobilisation reported the most reliable analytical recoveries. For example, the PEDOT/ILRGO platform retained 95.7% of its activity after 15–20 d and achieved 91.7% enzyme reactivation during analysis of apple juice [198], consistent with the greater resistance to enzyme leaching of covalent attachment through amino, carboxyl, or aldehyde functional groups compared with physical adsorption [120]. In contrast, sensors requiring stringent storage conditions, such as the PPy-IC-AuNP-AChE platform, were not evaluated using real samples, suggesting that their excellent analytical sensitivity has yet to be translated into demonstrated matrix tolerance [173]. Non-biological recognition strategies again showed favourable practical performance. The microfluidic MIP-PPy/Au microelectrode achieved recoveries of 91–97% in cucumber and pomegranate samples [114], reflecting the greater chemical robustness of synthetic recognition elements in complex matrices. Collectively, these observations indicate that successful real sample application depends less on achieving the lowest possible detection limit than on maintaining stable recognition chemistry and reliable sensor performance under realistic analytical conditions.

Overall, the comparative analysis presented in Table 9 demonstrates that superior CPC-based electrochemical sensors are not defined by a single conductive polymer or recognition strategy. Instead, their performances result from the synergistic optimisation of multiple structural and physicochemical properties. Consistently, across the representative studies, the most successful systems integrate heterostructured conductive architectures that promote rapid electron transfer kinetics [41,191], porous and well-dispersed morphologies that maximise electroactive surface area while minimising charge-transfer resistance [194,202], and robust recognition strategies that maintain analytical performance during prolonged operation and real sample analysis [114,120]. Rather than reflecting the intrinsic superiority of any one conductive polymer, the analytical differences observed across Table 9 are primarily determined by rational composite design, where the conductive polymer, secondary nanomaterial, and recognition element complement one another to overcome the limitations of each component. These comparative findings demonstrate that improvements in CPC-based electrochemical sensors arise principally from synergistic interfacial engineering, providing a mechanistic explanation for why certain composite materials consistently outperform others.

## 7. Challenges and Future Prospects

Despite the considerable progress detailed above, translation of CPC-based electrochemical sensors into routine environmental monitoring and food safety applications remains hampered by challenges. Importantly, not all these challenges are equally unresolved: Analytical sensitivity and detection limits have been substantially improved. However, reproducibility, long-term operational stability, matrix compatibility, and practical implementation continue to represent barriers to real-world deployment.

Sensitivity and detection limits can be regarded as challenges that have been largely addressed. Across the studies discussed in previous sections, femtomolar- to nanomolar-level detection limits have been repeatedly achieved for OPPs, including chlorpyrifos, parathion, and paraoxon, using PANI-, PPy-, PEDOT-, and PTh-based composites. The incorporation of carbon nanomaterials, metal oxides, MXenes, and noble metal nanoparticles has consistently enhanced electron transfer kinetics and electrocatalytic activity, enabling detection limits well below current regulatory thresholds. Consequently, further improvements in analytical sensitivity are unlikely to provide substantial practical benefits compared with addressing the challenges that continue to limit analytical reliability and field applicability.

Selectivity occupies an intermediate position. Real-world samples (agricultural runoff, industrial wastewater, fruits, vegetables, and other food matrices) are inherently complex and may contain numerous species that produce overlapping electrochemical responses or accelerate electrode fouling. MIPs, aptamer-based recognition elements, and enzymatic sensing strategies have significantly improved target specificity compared with earlier sensors, as discussed in Section 5.3. Nevertheless, cross-reactivity toward related OP analogues remains evident in even the most advanced sensing architectures discussed. Moreover, enzyme-based sensors continue to suffer from poor thermal stability, irreversible inhibition, and limited shelf life, motivating the development of non-enzymatic platforms based on CP nanocomposites, nanozymes, and affinity-based recognition interfaces.

Finally, reproducibility remains one of the least-resolved challenges and has received comparatively little systematic investigation. Many studies report excellent analytical performance using individually optimised, laboratory-fabricated electrodes, but fewer evaluate whether one reproduces a performance across independently fabricated sensors, different production batches, or separate laboratories. This issue extends beyond individual studies and reflects a broader challenge in electrochemical biosensor development, where analytical sensitivity is often prioritised over manufacturing consistency and metrological validation. The recent meta-analysis by Mascini et al. [203] evaluated 96 polymer-based biosensor studies published between 2015 and 2025 and found that approximately one-third failed to report reproducibility, while a similar proportion omitted precision measurements. Most studies estimated detection limits from idealised signal-to-noise ratios in buffer solutions rather than determining limits of quantification in the intended analytical matrices [203]. These findings reinforce the conclusion drawn from the literature reviewed here, namely that reproducibility remains substantially underreported despite the remarkable progress achieved in analytical sensitivity.

Several factors contribute to this persistent limitation. Electropolymerisation is highly sensitive to deposition parameters, including monomer concentration, scan rate, applied potential window, polymerisation charge, and electrolyte composition, all of which influence polymer thickness, morphology, conductivity, and active site distribution. Similarly, solution-based fabrication methods, such as drop-casting, frequently introduce variations in nanomaterial dispersion, solvent evaporation, and film thickness, resulting in electrode-to-electrode and batch-to-batch variability. Such inconsistencies affect background current, electroactive surface area, charge-transfer resistance, and overall analytical response, thereby limiting interlaboratory reproducibility and hindering the development of calibration-free disposable sensing devices. As emphasised by Nehru et al., the absence of standardised fabrication and validation protocols continues to impede regulatory acceptance and commercial translation of electrochemical pesticide sensors [204].

Long-term operational stability represents another challenge that has not advanced at the speed of analytical sensitivity. During prolonged electrochemical operation, CP films gradually undergo physicochemical degradation through processes such as overoxidation, dedoping, mechanical cracking, volumetric swelling, and partial delamination from the electrode surface. These changes progressively reduce electrical conductivity, electrocatalytic activity, and electron transfer efficiency, ultimately causing signal drift and analytical performance deterioration. In enzyme-based sensing platforms, additional instability arises from enzyme denaturation, cofactor leaching, irreversible inhibition, and loss of catalytic activity during storage and repeated measurements.

The importance of stability in electrochemical biosensing has long been recognised. Dhull et al. identified storage stability, sensitivity, and linear dynamic range as essential performance characteristics of acetylcholinesterase-based organophosphate biosensors [205]. Mascini et al. showed that operational stability remains inconsistently evaluated across polymer-based biosensor studies, with many reports either omitting shelf-life assessment or limiting stability evaluation to relatively short experimental periods [203]. Consequently, despite substantial advances in conductive polymer composite engineering, the long-term durability, regeneration capability, and operational lifespan of CPC-based sensors under realistic storage and field conditions remain insufficiently characterised. Addressing these issues must become a primary research priority if CPC-based electrochemical sensors are to progress from proof-of-concept laboratory devices to reliable analytical technologies.

Under practical analytical conditions, matrix effects further exacerbate these limitations. Variations in ionic strength, pH, buffering capacity, and the presence of coexisting electroactive compounds, including other pesticides, heavy metal ions, naturally occurring phenolic compounds, and organic matter, can significantly alter the electrochemical response of CPC-based sensors. In addition, humic substances, proteins, lipids, and other macromolecules commonly found in environmental and food samples readily adsorb onto electrode surfaces, resulting in surface passivation, electrode fouling, and progressive signal deterioration. Consequently, calibration curves established in standard buffer solutions often fail to predict sensor performance in complex matrices accurately, thereby reducing analytical accuracy and reproducibility during real sample analysis.

The studies summarised in Section 4 and Section 5 indicate that matrix effects remain insufficiently investigated despite the impressive analytical performances frequently reported for CPC-based electrochemical sensors. Most published studies evaluate sensor performance using buffer solutions or artificially spiked samples with relatively simple pretreatment procedures, whereas comprehensive validation using naturally contaminated samples is less common. Although recovery values obtained from spiked samples often fall within acceptable analytical ranges, recovery alone does not necessarily indicate matrix tolerance, as signal suppression, baseline drift, and adsorption-induced electrode fouling may still affect quantitative performance. Consequently, future studies should complement conventional recovery experiments with systematic investigations of matrix-induced signal variation, calibration stability, and long-term analytical performance across multiple environmental and food matrices.

These observations are consistent with the findings of Mascini et al., who identified insufficient validation in real samples as a principal limitation preventing the broader application of polymer-based biosensors. Their meta-analysis demonstrated that many studies continue to emphasise analytical sensitivity while providing comparatively limited evidence regarding quantitative performance under practical operating conditions, particularly in complex matrices where calibration uncertainty becomes increasingly significant [203]. Likewise, Nehru et al. emphasised that reliable pesticide monitoring requires sensor validation under realistic environmental conditions because laboratory-based analytical performance often overestimates practical applicability in heterogeneous agricultural and environmental samples [204].

Closely related to matrix compatibility is sensor-to-sensor variability, which becomes increasingly important as CPC-based electrochemical sensors move beyond proof-of-concept studies toward practical manufacturing. Minor variations in polymerisation conditions, composite composition, nanomaterial distribution, or electrode modification procedures may produce measurable differences in electrochemical behaviour, including changes in background current, charge-transfer resistance, peak current, and analytical sensitivity. Although such variations are generally acceptable during laboratory-scale optimisation, they become critical when reproducible performance is required across independently fabricated sensors or large production batches. Consequently, reducing sensor-to-sensor variability should be an integral part of sensor design rather than treated solely as a post-fabrication quality-control issue.

Addressing these challenges requires greater emphasis on fabrication standardisation and analytical validation alongside conventional material optimisation. Future studies should routinely report batch-to-batch reproducibility, interlaboratory validation, fabrication tolerances, and quality-control procedures using standardised experimental protocols. Such practices would facilitate meaningful comparisons among independently developed sensing platforms, increase confidence in reported analytical performance, and accelerate regulatory acceptance. As highlighted by Nehru et al., the absence of standardised validation procedures remains a major obstacle preventing the translation of electrochemical pesticide sensors from laboratory research to routine analytical applications [204].

Practical field implementation introduces additional challenges that remain relatively underexplored in the current literature. Many CPC-based sensors exhibit outstanding analytical performance under laboratory conditions, but relatively few studies address the engineering requirements necessary for routine field deployment. These include the development of portable, low-power potentiostats compatible with disposable screen-printed electrodes, robust reference electrode systems suitable for outdoor operation, scalable synthesis of advanced nanomaterials, and standardised validation procedures comparable to internationally accepted analytical methods. Furthermore, fabrication routes involving multiple nanomaterials, sophisticated surface modifications, or labour-intensive preparation steps may become difficult to reproduce consistently under industrial manufacturing conditions, thereby increasing production costs and limiting commercial scalability.

Encouraging progress has been achieved through the development of miniaturised electrochemical platforms integrating screen-printed electrodes with portable potentiostats, wireless communication modules, and smartphone-assisted data acquisition systems. As reviewed by Pérez-Fernández et al., screen-printed electrode technology provides a practical foundation for the fabrication of inexpensive, disposable, and portable electrochemical pesticide sensors suitable for on-site analysis [206]. More recently, Savas and Gharibzahedi highlighted the growing potential of smartphone-integrated electrochemical sensing platforms capable of combining wireless connectivity, simplified sample handling, cloud-based data management, and real-time analytical reporting for agricultural and food safety monitoring [207]. Nevertheless, these integrated systems have so far been demonstrated for only a limited number of CPC sensor architectures, and further efforts are required to combine advanced material performance with scalable device engineering and standardised analytical validation before widespread field deployment can be achieved.

Addressing these challenges will require advances that extend beyond conventional material optimisation. Continued improvements in conductive polymer chemistry and nanocomposite engineering remain important, but future progress will increasingly depend on integrating intelligent data analysis, scalable device engineering, and sustainable fabrication strategies. Rather than being viewed as independent research directions, these emerging approaches should be considered complementary technologies that can improve analytical robustness, reproducibility, and practical applicability.

Among these emerging technologies, artificial intelligence (AI) and machine learning (ML) have become particularly promising tools for enhancing electrochemical sensing performance. Unlike conventional signal-processing methods that rely primarily on peak height or peak current measurements, ML algorithms extract complex relationships from multidimensional electrochemical datasets, enabling improved analyte discrimination, baseline correction, drift compensation, and quantitative predictions. Consequently, AI-assisted electrochemical analysis offers a practical strategy for mitigating many of the unresolved challenges identified in this review, particularly matrix effects, signal instability, and sensor-to-sensor variability.

Current applications of ML in electrochemical sensing have progressed well beyond simple pattern recognition. As discussed by Giodarno, chemometric approaches such as principal component analysis and partial least squares regression have been successfully applied to deconvolute overlapping voltammetric responses, compensate for baseline drift, and improve quantitative analysis in complex matrices [208]. These methods provide an effective first step toward reducing analytical uncertainty associated with electrode fouling and matrix-induced signal distortion.

More advanced supervised learning algorithms have further expanded the analytical capabilities of electrochemical sensors. Support vector machines, random forests, and k-nearest neighbours have been employed to classify electrochemical fingerprints obtained from complex mixtures that would otherwise generate overlapping or ambiguous voltammetric responses. In particular, Dean et al. demonstrated that after recurrent neural networks and fully convolutional neural networks (CNNs) were trained using cyclic square-wave voltammetric data, they successfully discriminated between herbicides, pesticides, and structurally related compounds. These models achieved receiver operating characteristic area-under-the-curve values exceeding 0.99, outperforming several conventional ML classifiers [209]. These findings illustrate the considerable potential of deep learning for improving the analytical selectivity and classification accuracy of electrochemical pesticide sensors.

Deep learning has also shown promise for resolving a major analytical limitation identified in this review, namely cross-reactivity among structurally similar electroactive compounds. Molinara et al. converted cyclic voltammograms into Gramian Angular Field images and employed CNNs to distinguish hydroquinone and benzoquinone, two compounds exhibiting highly overlapping electrochemical responses. Their approach achieved substantially higher classification accuracy than conventional peak-based analysis, demonstrating that deep learning can effectively extract subtle electrochemical features that are difficult to distinguish using traditional analytical methods [210]. Similar approaches may provide an effective computational solution for improving the selectivity of CPC-based electrochemical sensors toward structurally related OPPs.

Beyond laboratory data analysis, ML is increasingly being incorporated into portable sensing systems designed for field deployment. Dhamu et al. integrated a portable potentiostat with an onboard ML classifier for the rapid categorisation of atrazine and glyphosate contamination in real soil samples, achieving prediction accuracies approaching 80% without the need for laboratory-based confirmation [211]. This study demonstrates that ML can be embedded directly within portable electrochemical devices rather than being limited to offline data processing. In the future, transfer learning and domain adaptation techniques may provide additional opportunities to compensate for batch-to-batch variability by recalibrating predictive models using only a limited number of new measurements, thereby reducing the extensive recalibration currently required when sensor fabrication conditions or sample matrices change [208,211].

Future CPC-based sensing platforms are therefore expected to integrate advanced conductive polymer composites with portable electrochemical instrumentation, wireless communication, cloud-based data management, and intelligent decision-support systems. The combination of disposable screen-printed electrodes, miniaturised potentiostats, smartphone-assisted signal processing, and embedded ML algorithms will enable rapid, user-friendly, and real-time monitoring of pesticide residues directly at the point of need. Such integrated platforms have the potential to support precision agriculture, environmental surveillance, and food safety monitoring while simultaneously improving analytical consistency and reducing dependence on centralised laboratory facilities.

In parallel with technological advances, increasing attention should also be directed toward the sustainability of electrochemical sensor development. Although significant efforts have been devoted to improving analytical performance, comparatively little emphasis has been placed on environmentally responsible sensor fabrication. Future research should therefore promote the use of renewable carbon sources, biodegradable substrates, green synthesis routes, low-toxicity reagents, and energy-efficient manufacturing processes. These approaches are consistent with the principles of green analytical chemistry and will become increasingly important as CPC-based electrochemical sensors move toward large-scale production and commercialisation.

As demonstrated throughout this review, analytical sensitivity has advanced substantially through innovations in CPCs and multifunctional nanomaterials. Overall, the future development of CPC-based electrochemical sensors should no longer be driven solely by the pursuit of increasingly lower detection limits. Instead, the next stage of development should prioritise reproducibility, long-term operational stability, matrix compatibility, standardised analytical validation, scalable manufacturing, and intelligent data processing. By integrating advances in materials science, electrochemical transduction, AI, and portable sensing technologies, CPC-based electrochemical sensors are well positioned to evolve from proof-of-concept laboratory devices into reliable analytical platforms capable of supporting routine environmental monitoring, food safety assurance, and sustainable agricultural management.

## 8. Conclusions

CPC-based electrochemical sensors have emerged as promising platforms for OPP detection because they combine favourable CP electrical properties with the complementary functions of nanostructured materials. Throughout this review, we have shown how conductive polymers, including PANI, PPy, PTh, and PEDOT:PSS, can be integrated with graphene-based materials, carbon nanotubes, metal oxides, MXenes, and MIPs to improve electron transfer efficiency, electroactive surface area, and molecular recognition. These composite architectures have consistently demonstrated improved analytical performance compared with their individual components, highlighting the importance of rational composite design for electrochemical pesticide sensing.

The development of CPC-based sensors has also expanded the range of available recognition strategies. Enzymatic biosensors continue to provide excellent molecular selectivity and high analytical sensitivity, whereas non-enzymatic approaches, including MIPs, aptamer-assisted platforms, and direct electrocatalytic sensors, offer improved operational stability, simplified fabrication, and greater suitability for long-term applications. Advances in flexible, paper-based, and wearable electrochemical devices have broadened the potential applications of CPC-based sensors for portable environmental and food safety monitoring.

Despite these advances, challenges remain before CPC-based sensors can be widely implemented in practice. Long-term operational instability, large-scale fabrication reproducibility, matrix interference in complex real samples, and lack of standardised validation protocols continue to limit the translation of many laboratory-scale sensors into routine field applications. Furthermore, additional studies evaluating long-term reproducibility, interlaboratory validation, and real-world performance under diverse environmental conditions are needed to establish the reliability of these sensing platforms.

Future research should therefore focus on the rational design of multifunctional CPCs that balance analytical sensitivity, selectivity, operational stability, and manufacturability. Continued integration of advanced nanomaterials with emerging approaches, such as artificial intelligence-assisted data analysis, flexible electronics, and sustainable fabrication strategies, may further improve sensor performance and facilitate practical deployment. Overall, CPC-based electrochemical sensors represent a promising direction for rapid and portable OPP monitoring, although continued technological development and comprehensive validation will be essential before their widespread adoption in routine environmental and food safety applications.

## Figures and Tables

**Table 1:** Physicochemical properties and toxicological profiles of key OPPs.

Pesticide Name	Chemical Formula	Molecular Weight (g mol^−1^)	Primary Use	Main Toxicity/Environmental Concern
Parathion	C_10_H_14_NO_5_PS	291.2	Broad-spectrum insecticide [56,65]	High neurotoxicity; chronic cognitive impairment; strictly regulated/banned [56,66]
Malathion	C_10_H_19_O_6_PS_2_	330.4	Agriculture, lice control, and mosquito control [67,68]	AChE inhibitor; moderate soil mobility/leaching potential [56,69]
Dichlorvos	C_4_H_7_Cl_2_O_4_P	221.0	Household, veterinary, and agricultural insecticides [66]	High volatility, potential carcinogenicity, and genotoxicity [66,70]
Fenitrothion	C_9_H_12_NO_5_PS	277.2	Sucking and chewing pest control [71]	Causes oxidative stress and hyperammonemia; respiratory risks [72,73]
Diazinon	C_12_H_21_N_2_O_3_PS	304.4	Veterinary medicine [74]	Non-target organism toxicity; potential environmental accumulation [74]
Chlorpyrifos	C_9_H_11_C_l3_NO_3_PS	350.6	Agricultural pest control [59,64]	Disrupts soil microbial communities; persistent residue in food/water [64]
Paraoxon	C_10_H_14_NO_6_P	275.2	Active metabolite/nerve agent [75]	Extreme toxicity; poses severe neurological and seizure risks [76]

**Table 2:** Physicochemical properties of CPs (PANI, PPy, PEDOT, and PTh).

Conductive Polymer	Typical Conductivity (S cm^−1^)	Primary Redox Characteristic	Functionalisation Capability	Electron-Transfer Characteristics	Key Advantage for OPP Sensing
PANI	10^−22^ to 10^2^	pH-dependent doping	High; amine groups allow direct chemical modification and click chemistry [98,99]	Doping-dependent; varies with oxidation state; [99]	Excellent redox reversibility [93]
PPy	10^−1^ to 10^2^	Stable oxidised state	Moderate; conductivity and morphology are both synthesis-dependent [100]	Stable across a wide conductivity range depending on doping and synthesis method [100]	Facile biomolecule immobilisation [101]
PEDOT	10^2^ to 10^3^	High stability/transparency	Low in pristine form; requires PSS doping for water processability [102]	High, though limited by structural disorder in the conjugated backbone [103]	Low bandgap, high carrier mobility [103]
PTh	10^−1^ to 10^2^	Excellent environmental stability	Moderate; side-chain functionalisation possible without loss of conductivity	Comparatively slower than PEDOT, but stable over time	Resistance to chemical degradation [104]

**Table 3:** Physicochemical properties of carbon-based conductive fillers (graphene, GO, rGO, SWCNT, MWCNT).

Carbon Filler	Conductivity Behavior	Stability	Functionalization Capability	Electron-Transfer Role
Graphene	Comparable to MWCNTs and carbon black in compacted form (~10^2^ S/m); intrinsic single-sheet conductivity is higher [125]	High, but prone to restacking in dispersion	Low; basal plane is largely chemically inert outside edge sites [126]	Fast in-plane transport through delocalized π electrons
Graphene oxide (GO)	Low; oxidation disrupts π conjugation [127]	High water solubility, lower structural order than graphene	High; abundant hydroxyl, carboxyl, and epoxy groups allow direct grafting [128]	Limited; conjugated pathway is broken by oxidation
Reduced graphene oxide (rGO)	Higher than GO, partially restored conjugation after reduction	Lower water solubility than GO	Moderate; fewer oxygen groups remain after reduction [128]	Improved over GO, though below pristine graphene
SWCNT/MWCNT	Conductivity comparable to graphene and carbon black in compacted form [125]	High mechanical and chemical stability	Moderate; both sidewall and end functionalization are possible	Efficient along the tube axis; commonly used as a bridge between other components

**Table 4:** Comparison of Enzymatic, Molecularly Imprinted Polymer-based, and Direct Electrocatalytic CPC Sensing Strategies.

Recognition Strategy	Selectivity	Operational Stability	Fabrication Complexity	Suitable Application Scenario
Enzymatic inhibition	Highest; molecular recognition originates from specific enzyme–substrate interactions [153]	Lowest; enzyme activity highly sensitive to pH, temperature, and irreversible inhibition, limiting long-term operational stability [111,112]	Highest; requires enzyme purification, immobilisation, and preservation of biological activity within conductive polymer matrix [96,154]	High-sensitivity laboratory analysis and confirmatory detection under controlled operating conditions
MIP	High; template-specific recognition provides excellent selectivity; structurally related analogues may still produce cross-reactivity [109,120]	High; chemically stable recognition cavities eliminate biological degradation; template leakage and incomplete template removal remain important limitations [114,120]	Intermediate; requires template synthesis, polymerisation, and template extraction but avoids biological handling and storage	Reusable field-deployable sensors for monitoring specific target pesticides
Direct electrocatalytic	Moderate to low; recognition depends on electrochemical oxidation or reduction of redox-active functional groups that may also be present in structurally related compounds [145]	Highest; absence of biological recognition elements minimises degradation and provides excellent long-term operational robustness once composite interface is stable [151]	Lowest; no enzyme immobilisation or template extraction required, resulting in relatively simple sensor fabrication	Rapid, portable, low-cost preliminary screening where fast analysis is prioritised over compound-specific identification

**Table 5:** Analytical Performance of PANI-Based CPCs Electrochemical Sensors for OPP Detection.

No	Electrode	Analyte	Method	Linearity	Sensitivity	LOD	Ref.
1	Cu_x_O-PANI/GCE	Chlorpyrifos	CV	1–12 µM	0.028 µA pM^−1^	0.54 pM	[155]
2	AChE/CeO_2_@PANI/ITO	Paraoxon-ethyl	DPV	1–100 pM	0.59 µA (pM)^−1^ cm^−2^	0.65 pM	[96]
3	MSO/PANI/GCE	Fenitrothion	CV, DPV	0.01–390 µM	0.43 μA μM^−1^ cm^−2^	0.46 nM	[156]
4	PANI/AuNPs electrode	Chlorpyrifos	Electrochemical biosensor	1.00 × 10^−3^–1.00 × 10^1^ ppm	N.A.	7.90 × 10^−5^ ppm	[157]
5	PANI/N-GQDs electrode	Profenofos	DPV	1 × 10^−1^–1 × 10^4^ ng mL^−1^	N.A.	3.59 ng mL^−1^	[158]
6	AChE/AgNPs/GO/PANI/SPCE	DMMP	CV	0.1–1 × 10^4^ ppb	N.A.	6.43 × 10^−5^ ppb	[159]
7	AChE/AgNPs/GO/PANI/SPCE	Omethoate	CV	0.1–1 × 10^4^ ppb	N.A.	1.07 × 10^−6^ ppb	[159]
8	MWCNT/PAnNF IDE	Organophosphorus pesticides	Resistive sensing	2.0–18.0 U mL^−1^	0.11 U mL^−1^ (AChE), 0.093 U mL^−1^ (BChE)	0.11 U mL^−1^	[85]
9	PANi/fMWCNT/ITO	Chlorpyrifos	CV	10–120 ng L^−1^	0.41 mA ng^−1^ L cm^−2^	8.8 ng L^−1^	[160]
10	PANi/fMWCNT/ITO	Methyl parathion	CV	10–120 ng L^−1^	0.58 mA ng^−1^ L.cm^−2^	10.2 ng L^−1^	[160]
11	MnPc-TA/N_3_-PANI/ITO	Fenitrothion	SWV	0.12–15.00 µM	4.67 A cm^−2^ M^−1^	0.049 µmol dm^−3^	[110]
12	MnPc-TA/N_3_-PANI/ITO	Diazinon	SWV	0.20–7.50 µM	9.15 A cm^−2^ M^−1^	0.062 µmol dm^−3^	[110]
13	Au/MBT/PANI/AChE/PVAc	Diazinon	Amperometric inhibition	N.A.	N.A.	0.147 ppb	[161]
14	Au/MBT/PANI/AChE/PVAc	Fenthion	Amperometric inhibition	N.A.	N.A.	0.172 ppb	[161]
15	Ag@CuO/PANI/ITO	Paraoxon-ethyl	Electrochemical detection	5–100 pM	0.5536 µA (pM)^−1^ cm^−2^	11.35 pM	[162]
16	PANI-PPy-MWCNTs/AChE	Malathion	Amperometry	0.01–0.5 μg mL^−1^ and 1–25 μg mL^−1^	N.A.	1.0 ng/mL	[163]
17	Au/PANI/MWCNTs/BDD	Parathion-ethyl	DPV, EIS	0.2–2 nM and 8–100 nM	N.A.	0.062 nM	[164]
18	TiO_2_-en-NC-g-PANI electrode	Malathion	Potentiometry	1.0 × 10^−12^–1.0 × 10^−6^ M	32.64 mV µM^−1^ cm^−2^	2.82 × 10^−9^ M	[165]
19	PANI-PVS-dsCT-DNA/ITO	Chlorpyrifos	SWV, EIS	0.5–100 ppb	N.A.	0.5 ppb	[166]
20	PANI-PVS-dsCT-DNA/ITO	Malathion	SWV, EIS	0.01–10 ppm	N.A.	0.01 ppm	[166]
21	AgNPs/MWCNTs/PANI/GCE	Fenitrothion	AdDPSV, CV, EIS	0.1–5.67 µg mL^−1^	N.A.	0.000193 µg mL^−1^	[167]
22	PANI film/ITO	Paraoxon	Amperometry	0.1–0.9 µM	N.A.	0.1 µM	[168]
23	MnO_2_/PANI/rGO/GCE	Methyl parathion	DPV, Amperometry	0.5–65 µM	3.05 µA µM^−1^ cm^−2^	7.4 nM	[169]
24	CuO/SA-g-PANI film	Chlorpyrifos	Potentiometry	1.0–120.0 µM	1.8790 mV µM^−1^ cm^−2^	0.375 µM	[170]
25	ZrO_2_/OMP electrode	Methyl parathion	SWV	5.96 × 10^−10^–1.02 × 10^−8^ M	N.A.	2.28 × 10^−10^ M	[171]
26	PANI-CdS/CeO_2_/Ag_3_PO_4_/GCE	Malathion	CV, EIS	1–13 × 10^−7^ M	N.A.	3.0 × 10^−9^ M	[172]

**Table 6:** Analytical Performance of PPy-Based CPC Electrochemical Sensors for OPP Detection.

No	Electrode	Analyte	Method	Linearity	Sensitivity	LOD	Ref.
1	PPy-IC-AuNP-AChE/FTO	Methyl parathion	CV	1.3 × 10^−7^–1.0 × 10^−3^ M	14 µA cm^−2^ g^−1^ mL	24 fmol L^−1^	[173]
2	AuNPs/PPy/HOOC-MWCNTs/GCE	Methyl parathion	Rapid scan CV	0.10–20.0 µM	N.A.	5.0 nM	[89]
3	CuO-in-CHIT-g-PPy electrode	Chlorpyrifos	Potentiometry	1–100 µM	2.44 mV µM^−1^ cm^−2^	0.75 µM	[174]
4	AChE-Chit/PPy/NiFe LDH/Ti_3_C_2_ MXene/GCE	Dimethoate	Biosensor	10^−10^–10^−4^ mol L^−1^	6.816 (% inhibition per −log C)	1.459 × 10^−11^ mol L^−1^	[175]
5	MXene/CNHs/PPy/AChE/GCE	Methyl parathion	DPV	0.002–346 ng mL^−1^	N.A.	0.00021 ng mL^−1^	[176]
6	MIP-PPy/PGE	Chlorpyrifos	EIS	20–300 µg L^−1^	N.A.	4.5 µg L^−1^	[177]
7	MIP-PPy/Au microelectrode	Chlorpyrifos	DPV/EIS	1 fM–1 µM	N.A.	0.93 fM	[114]
8	PPy/GCE	Profenofos	CV/DPV/EIS	1 nM–5 µM	N.A.	1 nM	[178]
9	CoNPs/PPy/GCE	Phoxim	SWV/CV/EIS	0.025–12 µM	N.A.	4.5 nM	[113]
10	dsCT-DNA/PPy-PVS/ITO	Chlorpyrifos	Amperometry/CV	0.0016–0.025 ppm	N.A.	N.A.	[179]
11	dsCT-DNA/PPy-PVS/ITO	Malathion	Amperometry/CV	0.17–5.0 ppm	N.A.	N.A.	[179]
12	IrOxNPs/PPy-MIP/ITO	Chlorpyrifos	Electrochromic sensing	100 fM–1 mM	N.A.	100 fM	[180]
13	Hg-PPy/GC	Methyl parathion, Methyl azinphos, Parathion, Fenitrothion	Fast linear sweep voltammetry	Detection at 1 ppm	N.A.	N.A.	[181]
14	PPy-AChE/Au	Paraoxon	Amperometry	N.A.	1.77 nA µM^−1^	N.A.	[182]
15	MIP-MEPs/GCE	Dimethoate	CV	0.1–1 nM	N.A.	N.A.	[183]

**Table 7:** Analytical Performance of Polythiophene-Based CPC Electrochemical Sensors for OPP Detection.

No	Electrode	Analyte	Method	Linearity	Sensitivity	LOD	Ref.
1	MIP poly(TAA-co-EDOT)/MWCNT/GCE	Chlorpyrifos	DPV	0.02–1000 nM	N.A.	4 × 10^−12^ M	[120]
2	PS2TTz/GCE	Chlorpyrifos	PEC	1–218.92 μg L^−1^	N.A.	0.36 μg L^−1^	[118]
3	Poly([2,2′;5′,2″]-terthiophene-3-carbaldehyde)/GCE	Malathion	Amperometry	0.015–1.644 mM	183.19 μA mM^−1^	4.08 nM	[121]
4	P3HT/TiO_2_/GCE	Chlorpyrifos	PEC	0.2–16 μM	High selectivity	0.01 μM	[117]
5	MnO_2_/PTH/rGO/GCE	Methyl parathion	CV, DPV, Amperometry	0.5–65 µM	0.0498 µA µM^−1^ cm^−2^	5.72 nM	[122]
6	PTh/TiO_2_/GCE	Malathion	CV	9.9–436 ppm	57.5 µA µM^−1^ cm^−2^	7.45 µM	[184]

**Table 8:** Analytical Performance of PEDOT and PEDOT:PSS-Based CPC Sensors for OPP Detection.

No	Electrode	Analyte	Method	Linearity	Sensitivity	LOD	Ref.
1	PEDOT:PSS screen-printed electrode/AChE	Chlorpyrifos-oxon	Amperometry (AChE inhibition)	N.A.	N.A.	4 × 10^−9^ M	[185]
2	PEDOT:PSS-MXene(Ti_2_CTx)-BSA-GO ISFET	Chlorpyrifos	P-ISFET electroreduction	1–4 µM	0.0279 µA s^−1^ µM^−1^	0.841 µM	[151]
3	Au-Ag core–shell graphene/PEDOT:PSS/GCE	Paraoxon-ethyl	CV, DPV, chronoamperometry	0.2–100 μM	3.24 μA μM^−1^ cm^−2^	10 nM	[145]
4	Pd-Fe_3_O_4_/PEDOT:PSS/NS-Ti_3_C_2_Tx	Parathion-methyl	Electroreduction	0.01–140 μM	517.08 μA mM^−1^ cm^−2^	3.3 nM	[186]
5	PEDOT:PSS/Zr(OH)_4_/ZIF-90	Parathion-methyl	CV, DPV	1–250 μmol L^−1^	0.09 μA μmol^−1^ L^−1^	0.74 μM	[187]
6	AChE/CuO@PEDOT:PSS/WP	Paraoxon-ethyl	Chronoamperometry	10–80 nM	9.1279 µA nM^−1^	0.42 nM	[188]
7	AChE/CuS@rGO/PEDOT:PSS/WP	Fenitrothion	Chronoamperometry	1–80 pM	0.505 mA pM^−1^	0.28 pM	[189]
8	W@Ce_2_MoO_6_/PEDOT:PSS/GCE	Fenitrothion	CV, DPV, EIS	0.066–136.66 μM	8.7142 μA μM^−1^ cm^−2^	6.6 nM	[190]
9	TiO_2_/CdS/PEDOT:PSS OPECT	Chlorpyrifos	Photoelectrochemical transistor	0.1 pg mL^−1^–100 ng mL^−1^	N.A.	2.2 fg mL^−1^	[191]
10	PEDOT:PSS/ITO	Paraoxon	LSV	N.A.	0.1767 A L mol^−1^	4.95 μM	[116]
11	AuNPs/Graphene-PEDOT:PSS/SPCE	Paraoxon	Amperometry (AChE inhibition)	10–400 ppb	0.1287% inhibition ppb^−1^	N.A.	[192]
12	PEDOT:PSS-rGO/AuNPs IDE array	Malathion, Cadusafos	Impedance spectroscopy (electronic tongue)	N.A.	N.A.	0.1 nmol L^−1^	[193]
13	CeO_2_@PEDOT:PSS conducting paper/AChE	Paraoxon-ethyl	Chronoamperometry	0.1–200 nM	52.10 μA nM^−1^	0.12 nM	[194]
18	PEDOT/GCE wall-jet electrode	Chlorpyrifos	SWSV	0.35–259.69 µg L^−1^	0.250 μA (μg L^−1^)	0.29 μg L^−1^	[195]
19	AChE/GP-AuNP-PEDOT:PSS/SPCE	Chlorpyrifos	DPV (AChE inhibition)	0.1–10 nM	3.69% inhibition nM^−1^	0.07 nM	[196]
20	ZrO_2_-chitosan/PEDOT/ITO	Methyl parathion	Electrochemical sensing	N.A.	N.A.	2.8 ng mL^−1^	[197]
21	PEDOT/rGO biosensor	Chlorpyrifos, malathion, methyl parathion	CV, DPV	N.A.	N.A.	0.04 ng mL^−1^	[198]
23	Ab/PEDOT-c-MWCNTs/FTO	Malathion	DPV	0.1 pM–1 μM	148 μA fM^−1^ cm^−2^	1.1 fM	[199]
24	PEDOT-f-MWCNTs/AChE	Chlorpyrifos-methyl	DPV, EIS	0.001–50 ppb	N.A.	0.001 ppb	[144]
25	AChE/PEDOT-MWCNTs/FTO	Malathion	CV, DPV	1 fM–1 μM	N.A.	1 fM	[154]

Note: Sensitivity values are presented as reported in original references. Where available, the lowest reported LOD and corresponding linear range are presented for comparison of analytical performance among PEDOT- and PEDOT:PSS-based sensing platforms for OPP detection.

**Table 9:** Comparative Analysis of Representative CPC-based Electrochemical Sensors for OPP Detection.

Polymer	Representative Composite	Target Analyte	Recognition Strategy	Electrochemical Technique	LOD	Stability	Real Sample Application (Recovery)	Key Advantages	Current Challenges	Ref.
PANI	Ag@CuO/PANI/ITO	Paraoxon-ethyl	AChE inhibition	DPV	11.35 pM	20 d storage (71.3% retained)	Banana, Tomato, Soil (92–111%)	Ag provides highest conducting power; bio-affinity via amino groups	Not reported	[162]
PANI	Au/PANI/MWCNTs/BDD	Parathion-ethyl	AChE inhibition	DPV, EIS, Amperometry	0.062 nM	30 d storage (94.6% retained)	Apple skin (90.1–99.4%)	Synergistic effects make electron transfer easier	Excess enzyme increases resistance/slows transfer	[164]
PANI	MnO_2_/PANI/rGO/GCE	Methyl parathion	Direct electrocatalysis	DPV, Amperometry	7.4 nM	30 d stable (RSD 2.34%)	Apple (84–90%), Milk (96–98%), Water	High catalytic ability for nitro group reduction	Not reported	[169]
PANI	TiO_2_-en-NC-g-PANI	Malathion	Potentiometric surface interaction	Potentiometry	2.82 × 10^−9^ M	64 d operational stability	Papaya pulp (100–102%)	Sustainable (rice husk-derived nanocellulose); long stability	Not reported	[165]
PANI	PANI/N-GQDs	Profenofos	Aptamer	DPV	3.59 ng/mL	28 d storage (7.6% current decrease)	Spinach, Oilseed rape, Lettuce (96–101%)	N-doping redistributes charge for higher conductivity	Acidic PANI conductivity vs. aptamer pH activity	[158]
PANI	AChE/PANi-fMWCNT/ITO	CPF & Methyl parathion	AChE inhibition	CV	8.8 ng/L (CPF); 10.2 ng/L (MP)	30 d shelf life (17% loss)	Cucumber (96.6–98%)	Simple two-step fabrication; nano-webbed structure	Enzyme degradation after prolonged storage	[160]
PPy	MIP-PPy/Au microelectrode	Chlorpyrifos	MIP	DPV	0.93 fM	70 d shelf life (11.8% loss after 70 d)	Cucumber, Pomegranate (91–97%)	Microfluidic cell (2 µL sample); ultrasensitive; label-free	Template removal and extraction time	[114]
PPy	MXene/CNHs/PPy/AChE/GCE	Methyl parathion	*AChE* inhibition	DPV	0.00021 ng/mL	15 d storage (88.6% retained)	Cabbage, soil, water (91.2–95.8%)	High loading capacity; solved MXene layer stacking	Loading hinders penetration; enzyme stability	[176]
PPy	PPy-IC-AuNP-AChE/FTO	Methyl parathion	AChE inhibition	CV	24 fmol/L	7 d @ −15 °C (84%)	Not reported	Increased sensitivity via AuNPs; smooth surface	Stability depends heavily on low-temperature storage	[173]
PPy	CoNPs/PPy/GCE	Phoxim	Direct electrocatalysis	SWV	4.5 nM	5 d storage (91% retained)	River water (94.6–100.2%)	Non-enzymatic; high electroactive surface area	Absorption of reduction products blocks sites	[113]
PPy	MIP/PPy/Pencil Graphite	Chlorpyrifos	MIP	EIS	4.5 μg/L	1 month storage (1.3% impedance loss)	Soil, corn leaves, water (101–103%)	Spongy structure provides high recognition sites	CPF metabolites (CPFO, TPD) interfere with response	[177]
PEDOT	TiO_2_/CdS/PEDOT:PSS OPECT	Chlorpyrifos	Chelation-induced photogating	Organic transistor (OPECT)	2.2 fg/mL	1440 min operational stability (no drift)	Not reported	Exceptional current gain (>800×); ultra-low noise	Complex device fabrication	[191]
PEDOT	AChE/PEDOT-MWCNTs/FTO	Malathion	AChE inhibition	Amperometry	1 fM	30 d storage; 7 times reusability	Lettuce (96–98%)	Fast electron transfer; high interface area	Matrix interference from lettuce blocks active sites	[154]
PEDOT	Apt/PEDOT-cMWCNTs/FTO	Malathion	Aptamer	DPV	0.5 fM	30 d storage (90% retained); 98% regeneration	Lettuce (95–100%)	High specificity; overcomes lack of specificity in AChE	Not reported	[200]
PEDOT	AChE/PEDOT/ILRGO/FTO	Malathion, CPF, Methyl parathion	AChE inhibition	DPV	0.04 ng/mL (CPF)	15–20 d (95.7% retained); 91.7% reactivation	Apple juice (1.03–2.74 ng/mL)	IL prevents stacking; increased hydrophilicity	Neat PEDOT has poor solubility/mechanical strength	[198]
PEDOT	CeO_2_@PEDOT:PSS Paper/AChE	Paraoxon-ethyl	AChE inhibition	Chrono-amperometry	0.12 nM	25 d storage (92.6% retained)	Soil (93–98%), Carrot (94–107%)	Flexible paper-based platform; EG reorients polymer	Enzyme dependence; paper layer diffusion	[194]
PTh	MIP poly(TAA-co-EDOT)/MWCNT/GCE	Chlorpyrifos	MIP	DPV	4.0 × 10^−12^ M	RSD 2.1% (repeatability); 25 cycles stable	Cucumber (97–98%); tap water (98–101%)	Specific binding sites on surface increase adsorption	Bulk imprinting slow kinetics (overcome via surface)	[120]
PTh	P3HT/TiO_2_/GCE	Chlorpyrifos	PEC	Amperometry (I-t)	0.01 μM	28 days (95.3% retained); RSD 4.1%	Green vegetables (consistent with GC/MS)	Visible light; zero potential sensing excludes matrix	Thick films prevent electron transport	[117]
PTh	MnO_2_/PTh/rGO/GCE	Methyl parathion	Direct electrocatalysis	DPV	5.72 nM	15 d stability (acceptable stability)	Human blood, serum, urine (88–97%)	High surface area and mobility of charge carriers	Not reported	[122]
PTh	Poly([2,2′;5′,2″]-terthiophene-3-carbaldehyde)/GCE	Malathion	AChE inhibition	CV, Amperometry	4.08 nM	10 d stability; RSD 1.7% repeatability	Parsley leaves (89.3–94.7%)	Covalent immobilisation via imine reaction	Response drops after 2 d without glutaraldehyde	[121]

## Data Availability

No new data were created or analyzed in this study. Data sharing is not applicable to this article.

## Abbreviations

The following abbreviations are used in this manuscript:

Ab	Antibody
AChE	Acetylcholinesterase
AdDPSV	Adsorptive Differential Pulse Stripping Voltammetry
AuNPs	Gold Nanoparticles
BChE	Butyrylcholinesterase
BSA	Bovine Serum Albumin
CP	Conductive Polymer
CPC	Conductive Polymer Composite
CV	Cyclic Voltammetry
DPV	Differential Pulse Voltammetry
EIS	Electrochemical Impedance Spectroscopy
FTO	Fluorine-Doped Tin Oxide
GCE	Glassy Carbon Electrode
GO	Graphene Oxide
IDE	Interdigitated Electrode
ISFET	Ion-Sensitive Field-Effect Transistor
ITO	Indium Tin Oxide
LDH	Layered Double Hydroxide
LOD	Limit of Detection
LSV	Linear Sweep Voltammetry
MIP	Molecularly Imprinted Polymer
MOF	Metal–Organic Framework
MXene	Two-Dimensional Transition Metal Carbide/Nitride Material
MWCNT	Multi-Walled Carbon Nanotube
OCV	Open-Circuit Voltage
OP	Organophosphate
OPH	Organophosphate Hydrolase
OPP	Organophosphate Pesticide
OPECT	Organic Photoelectrochemical Transistor
PANI	Polyaniline
PAnNF	Polyaniline Nanofiber
PEDOT	Poly(3,4-ethylenedioxythiophene)
PEDOT:PSS	Poly(3,4-ethylenedioxythiophene):poly(styrene sulfonate)
PEC	Photoelectrochemical
PGE	Pencil Graphite Electrode
PinCOOH	Poly(indole-5-carboxylic acid)
PPV	Poly(paraphenylene vinylene)
PPy	Polypyrrole
PSS	Poly(styrene sulfonate)
PTh	Polythiophene
P3HT	Poly(3-hexylthiophene)
PVS	Poly(vinyl sulfonate)
rGO	Reduced Graphene Oxide
SPCE	Screen-Printed Carbon Electrode
SWV	Square-Wave Voltammetry
SWSV	Square-Wave Stripping Voltammetry
Ti_3_C_2_Tx	Titanium Carbide MXene
ZIF-90	Zeolitic Imidazolate Framework-90
